# Rational Design of Mechanically Optimized Hydrogels for Bone Tissue Engineering: A Review

**DOI:** 10.3390/gels12010071

**Published:** 2026-01-13

**Authors:** Shengao Qin, Han Yuan, Zhaochen Shan, Jiaqi Wang, Wen Pan

**Affiliations:** 1 Department of Oral and Maxillofacial Surgery, School of Stomatology, Capital Medical University, Beijing 100070, China; shengaoqin123@163.com (S.Q.); shanzhch629@163.com (Z.S.); 2 Dental Medicine, School of Stomatology, North China University of Science and Technology, Tangshan 063210, China; 13373389862@163.com; 3 Department of Oral Medicine, School of Stomatology, Capital Medical University, Beijing 100070, China; jiaqiwang5923@163.com

**Keywords:** hydrogels, high-strength hydrogels, bone tissue engineering, bone repair, medical–engineering integration

## Abstract

Bone tissue engineering, as an important branch of regenerative medicine, integrates multidisciplinary knowledge from cell biology, materials science, and biomechanics, aiming to develop novel biomaterials and technologies for functional repair and regeneration of bone tissue. Hydrogels are among the most commonly used scaffold materials; however, conventional hydrogels exhibit significant limitations in physical properties such as strength, tensile strength, toughness, and fatigue resistance, which severely restrict their application in load-bearing bone defect repair. As a result, the development of high-strength hydrogels has become a research hotspot in the field of bone tissue engineering. This paper systematically reviews the latest research progress in this area: First, it delves into the physicochemical characteristics of high-strength hydrogels at the molecular level, focusing on core features such as their crosslinking network structure, dynamic bonding mechanisms, and energy dissipation principles. Next, it categorically summarizes novel high-strength hydrogel systems and different types of biomimetic hydrogels developed based on various reinforcement strategies. Furthermore, it provides a detailed evaluation of the application effects of these advanced materials in specific anatomical sites, including cranial reconstruction, femoral repair, alveolar bone regeneration, and articular cartilage repair. This review aims to provide systematic theoretical guidance and technical references for the basic research and clinical translation of high-strength hydrogels in bone tissue engineering, promoting the effective translation of this field from laboratory research to clinical application.

## 1. Introduction

Bone tissue engineering, as a frontier area of regenerative medicine, integrates multiple disciplines to develop functional repair systems based on biomaterials, cells, and bioactive molecules, with the goal of achieving precise regeneration of damaged bone tissue [1,2,3,4]. The core of bone tissue engineering lies in constructing a three-dimensional scaffold that can simulate the microenvironment of the natural extracellular matrix of bone cells. This scaffold not only provides temporary mechanical support but also regulates the adhesion, proliferation, differentiation, and osteogenic behavior of host or transplanted seed cells through its structure, composition, and functional modification. At the same time, an ideal scaffold should be biodegradable, and its degradation rate should match the growth rate of new bone tissue, ultimately achieving complete replacement by regenerated tissue and restoring the structural integrity and physiological function of the bone. A major challenge in this field lies in the complex interplay of numerous factors—such as host-related conditions [5,6], clinical variables [7,8], the local biological microenvironment [6,7,8,9,10], and the physicochemical properties of implanted materials [11]. Extensive studies have shown that the pore size and microarchitecture of tissue-engineered scaffolds greatly affect host cell adhesion, migration, aggregation, and functionalization [12,13,14,15,16]. Likewise, the mechanical properties of biomaterials are closely associated with bone regeneration efficiency [17,18,19], drug release kinetics [20], and inflammatory responses [21]. Thus, continuous optimization of scaffold design is essential for enhancing the regenerative performance of biomaterials within specific tissue environments.

Hydrogels are a class of hydrophilic polymer materials characterized by a three-dimensional (3D) network structure [22,23]. Their most remarkable feature is the ability to rapidly swell in water and retain a large amount of moisture without dissolving [24,25,26]. This high water content—often exceeding 90% of their volume—enables hydrogels to closely mimic the extracellular matrix (ECM) environment of human tissues, thereby providing an ideal three-dimensional niche for cell growth and migration [27,28,29]. However, the high water content also introduces several limitations, such as inappropriate degradation rates (either too rapid or too slow), insufficient permeability, and mismatched elastic modulus with native tissues. Among these, the most critical issue is the generally poor mechanical strength of hydrogels [17,30,31,32,33]. The elastic modulus of conventional hydrogels typically falls within the kilopascal (kPa) range, which is several orders of magnitude lower than that of natural bone tissue in the gigapascal (GPa) range. In addition, the tensile strength of traditional hydrogels is often below 100 kPa, while their compressive strength is usually less than 10 MPa—properties that can ultimately lead to repair failure [33,34,35]. To address these challenges, researchers have explored various strategies to enhance the overall mechanical performance of hydrogels.

The mechanical properties, biodegradability, and biocompatibility of bone repair hydrogels are closely interrelated and mutually influential, collectively determining the success of bone regeneration. An ideal hydrogel must balance mechanical performance with degradation kinetics, all while ensuring excellent biocompatibility. Specifically, its initial modulus should match that of bone tissue to provide stable mechanical support, and its degradation rate must align with the natural healing timeline, yielding only harmless, metabolizable byproducts. Current design approaches aim to harmonize these three aspects—for instance, by incorporating nanocomposites to enhance strength without compromising injectability, or by employing smart cross-links that respond to local biological cues to synchronize material degradation with new bone growth. The overarching objective is to achieve precise coordination between the hydrogel’s mechanical decline, release profile, and the physiological pace of bone regeneration.

This review systematically discusses the applications and recent advances of mechanically robust hydrogel-based biomaterials in bone tissue engineering. First, by comparatively analyzing the structural features and mechanical properties of high-strength hydrogels versus conventional hydrogels, the review highlights their remarkable advantages in bone repair applications. Second, based on differences in material composition and reinforcement mechanisms, existing high-strength hydrogels are scientifically classified, and the enhancement strategies and underlying mechanisms of each type are comprehensively analyzed. Third, drawing upon extensive in vitro and in vivo experimental data, the review provides a thorough evaluation of the bone repair efficacy of high-strength hydrogels in various defect models, offering evidence-based insights for their potential clinical applications. Finally, guided by biomimetic design principles, intelligent hydrogel systems with superior mechanical performance and biological functionality are proposed, and their future prospects in personalized bone tissue

## 2. Structure and Performance Characteristics of Traditional and Modified Hydrogels with Improved Mechanical Properties

**Figure 1:**
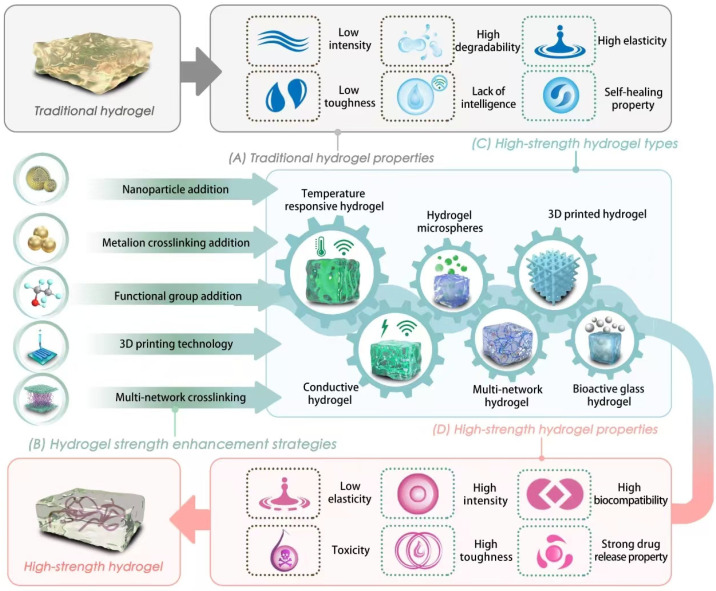
(**A**) Traditional hydrogels have the characteristics of high degradability, high elasticity, low strength, low toughness, insufficient intelligent responsiveness, and limited self-healing performance. (**B**) The strength of the hydrogel can be enhanced through the addition of nanoparticles, the introduction of multifunctional components, advanced structural design, and functional intelligence design. (**C**) High-strength hydrogels include types such as thermosensitive hydrogels, sensing hydrogels, hydrogel microspheres, multi-network hydrogels, 3D printed hydrogels, and bioactive glass hydrogels. (**D**) High-strength hydrogels exhibit high strength, high toughness, high biocompatibility, strong drug release properties, and low elasticity.

Conventional hydrogels are primarily composed of a single polymer network, in which the crosslinking points are fixed through permanent chemical bonds, resulting in a relatively simple network structure [36,37,38]. Although they possess a high water content and good flexibility, their mechanical strength is relatively poor. In particular, they tend to undergo brittle fracture under strong tensile or compressive stress, which significantly limits their applicability in load-bearing environments [38,39]. To overcome these shortcomings, researchers have developed a variety of hydrogels with superior mechanical properties. This section focuses on comparing the structural and physicochemical characteristics of conventional hydrogels with those of mechanically enhanced hydrogels, and further explores the underlying reasons for the mechanical deficiencies of traditional hydrogel systems (Figure 1A,D).

### 2.1. Traditional Hydrogels

The structural characteristics of conventional hydrogels are mainly represented by a single-network system, which is constructed from one type of polymer chain crosslinked through permanent covalent bonds to form a three-dimensional network structure [40]. In this configuration, the crosslinking points are typically irreversible and fixed, resulting in a uniformly distributed polymer chain spacing [41,42,43]. In terms of physicochemical properties, such hydrogels generally contain a high water content ranging from 70% to 99%, where most water molecules exist as free water rather than being effectively bound to the polymer chains. Although this feature endows the material with high softness and flexibility, it also leads to a significant reduction in mechanical strength [44,45,46]. From a mechanical standpoint, due to the simple single-network structure and immobile crosslinking points, stress tends to concentrate at localized regions under external load, thereby initiating crack propagation and resulting in typical brittle fracture behavior. Consequently, their tensile strength typically ranges from 10 to 100 kPa, which is far below the mechanical requirements of biological tissues such as bone and cartilage [46,47,48,49]. Moreover, conventional hydrogels exhibit poor elastic recovery, often displaying irreversible deformation after mechanical loading. The lack of an effective energy dissipation mechanism further hinders their ability to withstand repeated cyclic stresses [46,47,48,49,50,51]. From the perspective of chemical stability, the crosslinking points in traditional hydrogels are composed of permanent covalent bonds. Although this irreversible crosslinking ensures network stability, it also eliminates the material’s self-healing capability, thereby shortening its service lifespan [52]. Functionally, the static network formed by a single polymer matrix and permanent covalent bonds also restricts the controlled drug release performance of traditional hydrogels [53,54]. In addition, these materials exhibit pronounced environmental sensitivity; under strong acidic, alkaline, or high-salinity conditions, polymer chains and crosslinking points may degrade or dissociate, leading to a marked decline in both mechanical properties and structural stability [55].

### 2.2. Hydrogels with Excellent Mechanical Properties

Hydrogels with superior mechanical properties achieve a combination of high strength, toughness, and fatigue resistance through rational structural design, thereby greatly expanding their potential applications [56,57]. Toughness, an essential parameter reflecting a material’s ability to absorb energy and resist crack propagation, primarily arises from microscopic mechanisms such as reversible polymer chain slippage, energy dissipation within the crosslinked network, and the breaking and reformation of sacrificial bonds [58,59]. However, these mechanisms are often accompanied by significant energy dissipation, which may compromise the material’s elastic recovery capacity. Therefore, balancing toughness and elasticity is a crucial consideration in material design to meet specific application requirements. The elasticity of a hydrogel can be indirectly characterized by the ratio of the storage modulus to the loss modulus (i.e., the inverse of the loss factor) [60]. In bone tissue engineering, excellent elasticity not only ensures structural recovery and buffering capacity under cyclic physiological loading but also promotes mechanical matching and signal transmission between the implant and host tissue, thereby influencing osseointegration and long-term stability [61,62]. Tensile strength, as a core mechanical parameter, is mainly governed by the crosslinking density and the orientation of polymer chains [63]. Traditional high-water-content (>90%) hydrogels exhibit weak intermolecular interactions and sparse crosslinking networks, which reduce cooperative load-bearing among polymer chains and aggravate stress concentration [64,65,66]. Current research efforts focus on constructing high-strength hydrogels with optimized topological architectures. For instance, chemical double-network strategies that combine short-chain and long-chain crosslinking have been developed to synergistically enhance energy dissipation and elasticity retention, thereby achieving significantly improved strength and toughness [67,68]. However, high mechanical performance is typically associated with an increased crosslinking density, which, while improving structural stability, can reduce biodegradability [69]. To address this limitation, biodegradability can be enhanced by lowering the crosslinking density or incorporating degradable components and crosslinkers [70,71]. For example, polyurethane hydrogels synthesized using triol crosslinkers with varying chain structures have demonstrated a favorable balance between mechanical strength and degradability, thereby promoting bone tissue regeneration while minimizing secondary damage [72].

The chemical stability of hydrogels relies on their three-dimensional crosslinked network and the chemical inertness of their components, ensuring structural integrity under complex physiological conditions [73]. A representative example is the sodium alginate ionically crosslinked hydrogel fiber, which combines physical and ionic crosslinking mechanisms to achieve both high tensile strength and elongation at break, as well as excellent stability (tensile strength of 1.55 MPa and fracture strain of ~161%) [74]. It is worth noting, however, that the enhancement of mechanical strength in high-performance hydrogels often requires the introduction of additional crosslinkers or functional modifications, which may increase the risk of cytotoxicity [75]. Therefore, balancing mechanical enhancement and biocompatibility remains a key challenge in biomedical hydrogel design. Strategies to mitigate toxicity include the selection of naturally derived or medically approved polymers with superior biocompatibility, as well as optimization of formulation and fabrication processes [76,77]. A typical example is the bifunctional hydrogel membrane based on gelatin and hyaluronic acid, in which low-immunogenic gelatin provides cell adhesion sites, while hyaluronic acid contributes hydrophilicity and cell-regulatory functionality. This synergistic design effectively enhances the biocompatibility and osteoconductivity of the hydrogel [78] engineering are envisioned. Overall, this review aims to provide theoretical guidance for the development of novel bone repair materials, promote the clinical translation of hydrogel-based systems, and foster interdisciplinary integration and collaborative innovation across materials science and medicine. The characteristics of different types of hydrogels are compared in Table 1.

## 3. Strategies for Enhancing the Mechanical Properties of Hydrogels

### 3.1. Functional Responsive Hydrogels

Conventional hydrogels are primarily composed of water and polymeric materials, forming a relatively loose network structure that easily deforms or fractures under applied stress. As a result, they exhibit low mechanical strength and generally lack self-healing capability once ruptured [35]. In contrast, stimuli-responsive hydrogels represent a class of polymeric gels capable of responding to environmental cues such as temperature, pH, light, ionic strength, and electric fields, thereby undergoing volume phase transitions, shape-memory behavior, or reversible property changes [64,79]. By introducing crosslinkers, nanoparticles, or other reinforcement strategies, the mechanical strength of such hydrogels can be significantly enhanced, enabling them to withstand greater stress and deformation during bone defect repair [20,80,81]. Moreover, upon damage, these hydrogels can autonomously restore their structure through intermolecular interactions or dynamic chemical reactions, thereby improving their durability and reliability [82].

According to their distinct response mechanisms, smart hydrogels can be further categorized into temperature-sensitive, pH-responsive, photoresponsive, ion-responsive, and conductive hydrogels [83]. These hydrogels are capable of undergoing reversible structural transformations under specific environmental stimuli, a property that greatly enhances their functionality and adaptability, thereby allowing them to exhibit superior mechanical performance under designated conditions [84,85]. Moreover, through rational molecular design and the incorporation of functional components, the durability of such materials can be effectively improved, slowing down degradation processes and maintaining stable mechanical strength over extended periods [86]. For example, temperature-sensitive hydrogels can undergo volume or shape changes in response to temperature fluctuations. When the ambient temperature increases, these hydrogels typically expand or contract accordingly [87]. Several strategies have been developed to fabricate high-strength smart hydrogels. (1) Incorporation of nanoparticles. The introduction of nanoparticles into the hydrogel network increases the number of crosslinking points, thereby constructing a more stable and mechanically robust structure [88]. For instance, Yury et al. synthesized MXene using a mild approach and incorporated it via a two-step method into both self-healing HAPAM hydrogel networks and temperature-sensitive PNIPAM hydrogel networks. The resulting composite hydrogels exhibited excellent self-healing capability and thermal conductivity, along with enhanced mechanical strength. The hydrogel exhibits excellent mechanical properties, with a stretching ratio exceeding 14 times its original length, a tensile strength of up to 0.4 MPa, while maintaining good self-healing capability [89]. (2) Use of crosslinking agents. Incorporating an appropriate amount of crosslinker during hydrogel synthesis can also significantly improve mechanical properties [90]. For example, by employing vinyl-functionalized poly(N-isopropylacrylamide) (PNIPAM) microgels as macroscopic crosslinkers and copolymerizing them with *N*-isopropylacrylamide monomers, researchers successfully fabricated tough hydrogels with rapid response rates [91]. This bilayer-structured hydrogel not only demonstrated a markedly enhanced response rate but also exhibited excellent compressive strength. The improvement in mechanical performance was primarily attributed to the introduction of vinyl-functionalized microgels, which acted as macroscopic crosslinkers participating in reactions at two different polymerization stages. These crosslinkers formed network structures between polymer chains to enhance toughness, while simultaneously establishing internal crosslinking within the microgel domains, further strengthening the overall integrity of the hydrogel.

In addition, conductive hydrogels play a pivotal role in the field of fracture-healing monitoring [92]. Typically, conductive hydrogels are formed by incorporating conductive fillers—such as conductive polymers or carbon-based materials—into a polymeric matrix, resulting in composites with excellent electrical conductivity and sensitivity to environmental stimuli such as mechanical deformation and electrical signals [93]. However, the incorporation of conductive fillers often compromises the mechanical strength of hydrogels. Therefore, optimizing the synergistic interaction between the hydrogel matrix and the conductive components, while maintaining adequate conductivity, has become a key research focus in this field [94]. Recent studies have demonstrated that introducing ionic liquids or multivalent metal ions into hydrogel networks can create ionic or physical crosslinking structures, thereby enhancing the mechanical strength and energy dissipation capacity of the hydrogels [95,96]. For instance, Xu et al. successfully synthesized a novel conductive hydrogel—BSA-MA-PPy/P(AM-co-AA)/Fe^3+^—based on PPy-modified bovine serum albumin (BSA) and poly(acrylamide-co-acrylic acid) copolymers. Moreover, the hydrogel demonstrates exceptional overall performance, with a tensile strength of up to 5.36 MPa, toughness reaching 17.66 MJ/m^3^, and an elastic modulus of 1.61 MPa [97]. Through the combination of a conductive conjugated polymer (PPy) and metal–ligand coordination, the hydrogel exhibited high mechanical strength and rapid self-recovery properties. Benefiting from their unique mechanical robustness and electrical responsiveness, high-strength conductive hydrogels can accurately and continuously sense pressure variations at fracture sites and convert them into electrical signals. This functionality provides clinicians with real-time, quantitative data for assessing the progression of bone healing [94].

The development of stimuli-responsive hydrogels effectively addresses the complex requirements for bone regeneration, with biocompatibility being a crucial factor for their successful clinical translation. For example, hydrogels incorporating active ions (e.g., silver ions) have demonstrated enhanced wound healing in rodent models. However, precise control over ion release is essential to prevent potential local cytotoxicity. Additionally, pH-responsive silver hydrogels have been shown to accelerate wound healing in rat models of infected wounds, without inducing systemic toxicity or local inflammation. These findings underscore the importance of controlled release of bioactive ions to ensure the safety of the hydrogels. Collectively, these studies highlight that rationally designed stimuli-responsive hydrogels can exhibit favorable safety profiles in animal models, supporting their potential for clinical applications [98].

### 3.2. Hydrogel Microspheres

Hydrogel microspheres are spherical structures with micrometer-scale diameters fabricated from hydrogel materials. The mechanical strength of hydrogel microspheres is closely related to the crosslinking density of their internal polymer networks [99]. A higher crosslinking density results in a more compact network structure and, consequently, greater mechanical strength [100]. In addition, the size of the microspheres also influences their mechanical properties. Smaller hydrogel microspheres generally exhibit relatively higher mechanical strength due to their more uniform network architecture and reduced local stress concentration effects [101]. Several commonly employed strategies have been developed to enhance the mechanical performance of hydrogel microspheres: (1) Use of multifunctional crosslinkers: Incorporating bifunctional or multifunctional crosslinkers increases the number of crosslinking points and the structural complexity of the polymer network, thereby improving mechanical strength [102]. (2) Increasing crosslinker concentration: Elevating the concentration of crosslinking agents enhances the crosslinking density among polymer chains, leading to improved rigidity and strength [103]. (3) Incorporation of inorganic nanofillers or reinforcing polymers: Introducing inorganic nanoparticles (e.g., SiO_2_, graphene oxide) or high-strength polymers (e.g., PVA, PAM) into the hydrogel microspheres forms nanocomposite networks. Interfacial interactions between the fillers and the polymer matrix reinforce the network structure, enhancing both mechanical strength and energy dissipation capacity [104]. (4) Metal ion crosslinking: Incorporating multivalent metal ions (e.g., Ca^2+^, Al^3+^) can form ionic bonds with negatively charged groups within the hydrogel matrix, thereby strengthening the microspheres [105]. Such ionic crosslinks are typically strong yet reversible, making them particularly suitable for bone tissue engineering applications. (5) Introduction of functional groups with strong intermolecular interactions: Incorporating functional moieties capable of hydrogen bonding or hydrophobic interactions strengthens interchain associations and further enhances the mechanical integrity of hydrogel microspheres [106].

### 3.3. Three-Dimensional Printed Hydrogels

Three-dimensional printing technology, an additive manufacturing method based on a layer-by-layer deposition process, enables the fabrication of three-dimensional structures through the controlled stacking of materials [107]. In terms of mechanical performance, 3D printed hydrogels exhibit several distinctive characteristics: (1) Controllable mechanical properties. Three-dimensional printing allows for precise control over the geometry and internal architecture of hydrogels, enabling the mechanical strength to be tailored through design optimization [108]. Parameters such as layer thickness, printing speed, and printing path can be fine-tuned to modulate the overall mechanical behavior of the printed constructs [109]. (2) Anisotropy. Due to the layer-by-layer fabrication process, 3D printed hydrogels often exhibit anisotropic mechanical properties [110]. The strength along the printing direction may differ from that perpendicular to it. This anisotropy can either be minimized or strategically exploited through adjustments in printing parameters and structural design [110]. (3) Multi-material printing. Three-dimensional printing enables the integration of multiple materials, including various hydrogels and reinforcing components [111]. By combining high-strength materials with hydrogels, composite structures with enhanced mechanical performance can be fabricated, exhibiting region-specific or direction-dependent mechanical reinforcement [112].

Beyond structural design, several fabrication strategies have been developed to further enhance the mechanical properties of 3D printed hydrogels. First, the layered impregnation method has been utilized, wherein poly(vinyl alcohol) (PVA) hydrogels are incorporated into porous bioceramic scaffolds via the freeze-assisted solution substitution (FASS) process combined with a tannic acid (TA)–glycerol system. This synergistic approach significantly improves the mechanical stability and structural integrity of 3D printed scaffolds [113]. The method facilitates effective fiber bridging between different hierarchical layers and establishes hydrogen bonding among hydrogels and between hydrogels and bioceramics, resulting in a multi-reinforced architecture with superior mechanical robustness [113]. Second, low-temperature printing technology can be employed to strengthen hydrogels during fabrication. Zhao et al. developed a custom low-temperature 3D printer with a self-compiled G-code printing path, inspired by the hierarchical microstructure of mantis shrimp dactyl clubs. The printed PVA–LS and PVA–CMC hydrogels featured layered variable-angle architectures, achieving exceptional mechanical strength [114]. Third, electromagnetic field (EMF)-assisted printing has been explored to produce hydrogels with enhanced mechanical properties and improved printability [115]. Sayan et al. demonstrated that under low-frequency, low-intensity EMF conditions (5 V–1 Hz, 0.62 mT), 3D bioprinted thermosensitive Pluronic F127 (poly(ethylene oxide)–poly(propylene oxide) copolymer) hydrogels exhibited significantly enhanced osteogenic differentiation of stem cells from the apical papilla (SCAP) [115].

### 3.4. Multi-Network Hydrogels

Conventional hydrogels are primarily composed of single-network hydrophilic polymers, typically characterized by non-uniform crosslinking densities, resulting in low fracture energy and high brittleness [116]. In contrast, double network (DN) hydrogels are a class of materials formed by interpenetrating two or more polymer networks with distinct physicochemical properties [117]. This structural design aims to significantly enhance the mechanical performance of hydrogels—particularly their tensile strength, toughness, and fracture resistance. Generally, multi-network hydrogels are categorized into double network (DN) and triple network (TN) hydrogels [118].

A double network hydrogel typically consists of one rigid (brittle) network and one flexible network. The rigid network, often composed of highly crosslinked or brittle polymers, provides the hydrogel with high strength [119,120]. Because this network tends to fracture under strain, it dissipates a substantial amount of mechanical energy during deformation, effectively preventing the propagation of cracks [121]. The secondary network is usually composed of low-modulus, highly stretchable polymers that can bear stress and maintain the structural integrity of the hydrogel after the primary network undergoes partial rupture, thereby preventing catastrophic failure. The presence of this flexible network allows the hydrogel to deform under stress without fracturing [122]. Such a synergistic network design effectively distributes applied stress—an especially desirable feature in bone tissue engineering—and results in a substantial improvement in mechanical strength. Common fabrication methods for DN hydrogels include one-step polymerization, two-step polymerization, and post-crosslinking treatment [123,124]. Triple network (TN) hydrogels, developed as an advanced form of multi-network hydrogels, introduce a third polymer network to further enhance the mechanical strength, functional diversity, and structural stability of the material [118,125]. Compared with DN hydrogels, the third network in TN systems is often designed to fine-tune elasticity or impart additional functionalities. This tertiary network may consist of an elastomeric polymer, a highly crosslinked structure, or a functional polymer network. The preparation of TN hydrogels generally involves stepwise crosslinking, one-pot polymerization, or post-treatment methods [126]. In weight-bearing applications such as femoral repair, triple-network (TN) hydrogels exhibit superior fatigue resistance compared to double-network (DN) hydrogels. The fatigue threshold of TN hydrogels is 2.5–3.2 MPa·m^1^/^2^, whereas that of DN hydrogels is 0.8–1.2 MPa·m^1^/^2^. This difference arises from the synergistic energy dissipation and crack arrest mechanisms of the multiple crosslinking networks in TN hydrogels. However, DN hydrogels offer advantages in terms of simpler preparation and lower cost. The fatigue resistance of both types is significantly influenced by the crosslinking type, network interlocking, and crosslinking density. When the stress retention requirement is >90%, DN hydrogels such as PVA/TA-Fe^3+^ hydrogels can withstand only 30–50 cycles, while TN hydrogels such as PVA/PAAm/CNFs hydrogels can endure 200–500 cycles, which is 4 to 16 times higher than DN hydrogels, making TN hydrogels more suitable for applications like femoral repair that involve long-term repetitive loading [127].

### 3.5. Bioactive Glass Hydrogels

Bioactive glass (BG) is a class of biologically active amorphous silicate ceramics capable of promoting bone tissue growth and osseointegration by forming a bone-bonding interface [128]. Consequently, BGs hold great potential for restoring diseased or damaged bone to its original structure and function, thereby achieving true bone regeneration. In addition to their bioactivity, BGs exhibit a mechanical reinforcement effect—namely, the mechanical strength of hydrogels tends to increase with the incorporation of BG particles. However, simply mixing BG with hydrogel matrices does not fully exploit the advantages of either component [129]. Therefore, the method by which BG is incorporated into hydrogels is a critical factor deserving thorough discussion.

The first approach is the sol–gel method, in which BG precursors undergo hydrolysis and condensation reactions to form a sol that is subsequently co-crosslinked with hydrogel precursors, resulting in a homogeneous composite network gel [130]. This technique not only ensures the uniform distribution of bioactive glass sol within the hydrogel but also allows for the control of composition and structure at the molecular level, thereby maintaining the original chemical and phase structures of the bioactive glass. Moreover, because BG nanoparticles typically exhibit poor dispersibility, the sol–gel process can also improve their dispersion within the matrix [131,132]. For instance, Chen et al. synthesized Ce-doped bioactive glass nanoparticles (Ce-BG NPs) using a combined sol–gel and templating method, and subsequently incorporated them into gelatin methacryloyl (GelMA) hydrogels to fabricate a multifunctional injectable composite hydrogel [133]. The second approach involves crosslinking reactions. Surface-modified BG can form covalent or coordination bonds with hydrogel precursors, establishing a stable crosslinked network in which BG particles are firmly immobilized [134]. Compared with the sol–gel method, this strategy provides stronger interfacial bonding and greater structural stability. For example, researchers have employed silane coupling agents such as (3-aminopropyl)triethoxysilane (APTES) to introduce amino functional groups onto the surface of BG nanoparticles (BGNs), yielding amino-functionalized BG nanoparticles (ABGNs) with enhanced covalent bonding affinity to polymer matrices [135]. Hydrogels incorporating bioactive glass exhibit excellent biocompatibility, antibacterial activity, mechanical strength, and osteogenic capacity, making them highly promising for a wide range of applications in bone tissue engineering, including materials for bone regeneration and defect repair [136]. In conclusion, for different types of hydrogels, the mechanical strength of hydrogels can be enhanced through a series of methods to meet various requirements (Figure 1B,C).

## 4. Applications of High-Strength Hydrogels in Bone Tissue Engineering

### 4.1. Cranial Bone Tissue Engineering

Cranioplasty materials must satisfy several critical requirements. First, due to the typically large size of cranial defects, the selected material should exhibit high mechanical strength and cost-effectiveness to ensure structural stability and clinical feasibility. Second, given the complex intracranial environment, the material should possess a low swelling ratio to prevent increased intracranial pressure. Finally, excellent biocompatibility and interfacial adhesion are essential to ensure seamless integration with the remaining skull bone [137]. In these aspects, high-mechanical-performance hydrogels have demonstrated considerable potential as cranioplasty materials [138]. Studies have shown that inorganic hydrogels based on bioactive glass (ABG) [139], Ca^2+^-chelating hydrogels fabricated via Michael addition layered scaffolds [140], and multi-network hydrogels [141] can all effectively promote cranial bone regeneration. Notably, the stiffness of hydrogels plays a crucial role in regulating osteogenic activity—hydrogels with higher mechanical strength tend to exhibit superior bone-forming capabilities. Moreover, antibacterial nanocomposite hydrogels that integrate both antimicrobial and osteogenic functionalities, along with enhanced mechanical strength, offer additional advantages by preventing postoperative infections and improving the stability of newly regenerated bone [142]. For instance, silver nanoparticle-incorporated hydrogels not only inhibit microbial infection but also improve mechanical robustness and osteogenesis [142]. Similarly, magnesium-modified hydrogels [143] and silver-functionalized biomimetic antibacterial hydrogels [144] exhibit comparable effects, maintaining a sterile surgical environment while reinforcing the hydrogel’s mechanical integrity. These materials also function as barrier membranes, preventing endothelial and fibroblast invasion into the defect region.

Another key property is the anti-swelling capability of hydrogels. High-strength hydrogels can effectively regulate intracranial pressure and prevent cerebral edema. Studies have shown that divalent anion-crosslinked hydrogels [145] and injectable dual-network hydrogels composed of rigid and flexible polymers [146] can significantly enhance both mechanical properties and swelling resistance, while simultaneously promoting cranial bone regeneration. Furthermore, high-performance hydrogels can act as supportive scaffolds that promote angiogenesis by facilitating cell–cell interactions, thereby accelerating bone healing. For example, Hu et al. achieved vascularized cranial bone regeneration using hydrogel-coated scaffolds [147], whereas Chen et al. incorporated inorganic silica frameworks into hydrogels to endow them with superior mechanical strength, providing a favorable microenvironment for vascular network formation and new bone deposition within the defect area [148]. In summary, mechanically robust hydrogels, by simultaneously enhancing osteogenic potential, antibacterial activity, and anti-swelling capability, represent a highly promising class of biomaterials for cranial defect repair. These hydrogels not only promote efficient cranial bone regeneration but also help reduce postoperative complications [149].

### 4.2. Femoral Bone Tissue Engineering

As the primary load-bearing bone in the human body, the femur is subjected to complex mechanical stresses, and its cortical bone exhibits high compressive strength and elastic modulus [150]. Therefore, an ideal femoral repair material must possess high compressive strength, an appropriate elastic modulus, and fatigue resistance [151]. Compared with conventional hydrogels, recent studies have proposed novel fabrication strategies to enhance hydrogel mechanical performance for femoral tissue engineering applications. Strategy I: Nanoreinforcement. Incorporation of nanoparticles can significantly improve hydrogel mechanical strength and toughness. For example, Chen et al. integrated hydroxyapatite (HA) and magnesium oxide (MgO) nanocrystals into a hydrogel network, enhancing mechanical performance while promoting osteogenic differentiation of bone marrow mesenchymal stem cells. This nanocomposite hydrogel also exhibits immunomodulatory and proangiogenic functions, making it suitable for load-bearing bone defect repair [152]. Daniela et al. developed an injectable, photoresponsive nanocomposite hydrogel by combining graphene oxide (GO) with chitosan, achieving both mechanical stability and excellent biocompatibility [153]. Ismat et al. fabricated Sr/Fe-doped hydroxyapatite–collagen nanocomposite hydrogels with drug-loading capability, demonstrating dual osteogenic and antibacterial functions, thereby showing potential for the treatment of infectious femoral defects [154]. Strategy II: Multinetwork structures. Formation of hierarchical three-dimensional (3D) crosslinked networks can markedly enhance hydrogel mechanical strength and stability. Wang et al. designed a noncovalently crosslinked hydrogel exhibiting self-heating and shear-thinning properties, effectively preventing defect collapse while promoting osteoblast proliferation [155]. Noshad Peyravian constructed a 3D network via dynamic covalent bonding between aldehyde and amino groups, achieving a balance between mechanical performance and injectability, while facilitating angiogenesis and bone regeneration [156]. Liu et al. employed chemical crosslinking combined with carbon nanomaterial doping to enhance hydrogel mechanical properties and conductivity, improving preosteoblast adhesion and differentiation [157]. Zhang et al. fabricated a stable double-network hydrogel through freeze–thaw cycling, ionic crosslinking, and enzymatic mineralization, exhibiting excellent mechanical performance and bone regenerative capacity. The 3D porous double-network structure maintains a stable osteogenic environment, yielding smaller and more uniformly distributed mineralized particles, ultimately achieving efficient femoral regeneration. Strategy III: Scaffold-assisted reinforcement. Overall mechanical performance can be further improved by incorporating load-bearing structures [158]. For instance, Qin et al. utilized a spring-shaped poly(lactic-co-glycolic acid) (PLGA) scaffold to provide mechanical support for hydrogels, enhancing antibacterial and osteogenic properties while retaining suitability for minimally invasive injectable applications [159].

### 4.3. Alveolar Bone Tissue Engineering

Periodontitis is a chronic bacterial infectious disease in which bacterial toxins stimulate the gingiva, causing prolonged inflammation that ultimately leads to periodontal pocket formation, tooth mobility, and alveolar bone loss [160]. Since the alveolar bone bears most of the masticatory force, ideal repair materials must exhibit antibacterial and anti-inflammatory properties as well as sufficient compressive strength to maintain bone stability and promote bone healing [161]. Additionally, materials should allow minimally invasive, precise filling of defect sites to optimize repair outcomes [162]. Current alveolar bone tissue engineering often employs a “two-in-one” strategy, enhancing hydrogel mechanical strength while incorporating antibacterial components or modulating the local microenvironment to inhibit bacterial growth and promote osteogenesis [163]. For example, Shuo Xu et al. crosslinked chitosan (CS) with antimicrobial peptide-modified polyethylene glycol to form a dual-antibacterial hydrogel loaded with curcumin nanoparticles. The thiol groups in CS and maleimide groups underwent thiol–Michael addition reactions, resulting in a hydrogel that maintained solid-like characteristics and high mechanical strength after gelation, thereby providing long-term anti-inflammatory effects and robust structural support [164]. Junyu Liu synthesized glucose-sensitive high-strength hydrogels using tannic acid (TA)-modified chitosan, and the tensile strength and elastic modulus of the TA-incorporated films were significantly higher than those of pure CM hydrogel films. The modified hydrogel exhibited excellent antibacterial and anti-inflammatory activity while promoting periodontal tissue regeneration [165]. Recent studies have further shown that incorporating iodine-loaded PLGA–collagen fragments into an oxidized hyaluronic acid matrix, followed by catechol–Fe^3+^ coordination and Schiff-base crosslinking, enhanced the hydrogel’s structural integrity, injectability, and mechanotransduction capabilities. This design stabilized tension, activated the PIEZO1–ITGα5 signaling pathway, modulated immune responses, and promoted osteogenic differentiation [166].

Given the complex anatomy of periodontal pockets and defect morphology, minimally invasive injection has become the preferred delivery method. However, hydrogels must balance high mechanical strength with injectability. To achieve this, researchers have optimized hydrogel performance by adjusting crosslinking density, introducing nanomaterials, and incorporating low-viscosity high-strength polymers. For instance, Chang developed an LED-crosslinked collagen hydrogel with sufficient mechanical strength to support cell growth. Exposure to LED light can increase the tensile strength of collagen scaffolds. This result suggests that the hardened collagen scaffolds help periodontal ligament fibroblasts (HPLFs) maintain their relative positioning and release regulatory factors, thereby enhancing the effectiveness of periodontal regeneration [167]. Yu et al. fabricated a multifunctional injectable hydrogel containing MXene nanosheets and PL peptides, exhibiting anti-inflammatory, antibacterial, and osteoconductive properties, demonstrating excellent potential for periodontal tissue regeneration [168]. Xu et al. prepared an injectable polysaccharide hydrogel containing nano-hydroxyapatite (nHA) that enhanced mechanical performance, supported osteogenesis, and inhibited microbial growth, addressing challenging periodontal bone defects [169]. Moreover, periodontal therapy often faces short drug half-lives and the need for repeated administration [170]. High-strength hydrogel systems can achieve long-term drug release through several strategies, reducing patient burden: Dual-layer adhesive films have been developed to co-deliver moxifloxacin and clove oil, providing immediate and sustained antibacterial effects. Fold endurance tests demonstrated both flexibility and high mechanical strength [171]. Sequential dual-drug release via drug–matrix interactions. Xu designed CS/β-GP/gelatin hydrogels to sustain release of aspirin and erythropoietin (EPO) for up to 21 days. At temperatures above 37 °C, the pre-gel underwent sol–gel transition, achieving tensile strength up to 50.7 kPa (strain 76%), fully meeting the mechanical requirements for periodontal scaffolds [172]. Feng introduced dynamic boronate ester bonds and incorporated EGCG to enhance mechanical strength while enabling microenvironment-responsive drug release. Despite these advances, precise control over drug release rates and optimization of hydrogel degradation remain major challenges, directly impacting the safety and efficacy of clinical applications [173].

### 4.4. Articular Cartilage and Bone Tissue Engineering

Articular joints are composed of cartilage, subchondral bone, and synovial membranes, serving as essential functional units of the human body that bear mechanical stress during movement. Diseases such as osteoarthritis (OA) can lead to cartilage degeneration, osteophyte formation, and loss of mechanical function [174]. Due to the avascular and aneural nature of cartilage, its regenerative capacity is extremely limited, and effective repair of osteochondral defects requires materials that provide both mechanical robustness and layered functionality [175]. Insufficient mechanical strength in repair materials may result in delayed healing, exacerbated pain, and joint deformities.

In subchondral bone and cartilage repair research, numerous innovative materials have been developed, typically featuring: (1) composite network architectures to optimize mechanical properties; (2) precision 3D printing to construct biomimetic scaffolds; and (3) bioactive functionalities that regulate tissue regeneration. For instance, Li’s team developed a double-network hydrogel scaffold combining a rigid, brittle primary network with a soft, ductile secondary network, enabling sustained release of exogenous factors to promote cartilage and bone repair [176]. Jiang et al. fabricated PRP–GelMA composite scaffolds using DMD-based 3D printing technology. These scaffolds exhibited excellent mechanical properties and enhanced mesenchymal stem cell (MSC) proliferation, differentiation, and M2 macrophage polarization, creating a restorative microenvironment [177]. Lv’s team improved the mechanical properties of FEK hydrogels by incorporating carbon nanotubes and integrated them with 3D printed polycaprolactone (PCL) scaffolds to form a composite system, effectively promoting cartilage and subchondral bone regeneration in a rabbit knee defect model [178]. Drug delivery strategies are equally critical. Conventional oral or intravenous administration often suffers from low bioavailability and systemic side effects, whereas intra-articular injection can overcome these limitations, minimize patient discomfort, and rapidly fill irregular defects, thereby enhancing drug delivery efficiency. For example, Fang designed an injectable oxidized sodium alginate/gelatin/chondroitin sulfate (OSAGC) hydrogel with a dynamic covalent double-network structure and self-heating properties, promoting bone tissue regeneration within four weeks [179]. Cao developed a collagen–chondroitin sulfate biomimetic bilayer injectable hydrogel with strong interfacial adhesion and zinc-doped hydroxyapatite, facilitating subchondral bone regeneration [180]. Ji constructed a “building block” drug delivery system comprising porous chitosan (CS) microspheres embedded in thermosensitive hydroxypropyl methacrylate hydrogels. The highly stable CS microspheres supported endochondral ossification and subchondral bone regeneration, while the hydrogel matrix provided sustained mechanical support for drug delivery [181]. The relevant experimental methods and results are briefly presented in Table 2.

## 5. Strategies for Preparing High-Strength Biomimetic Hydrogels

**Figure 2:**
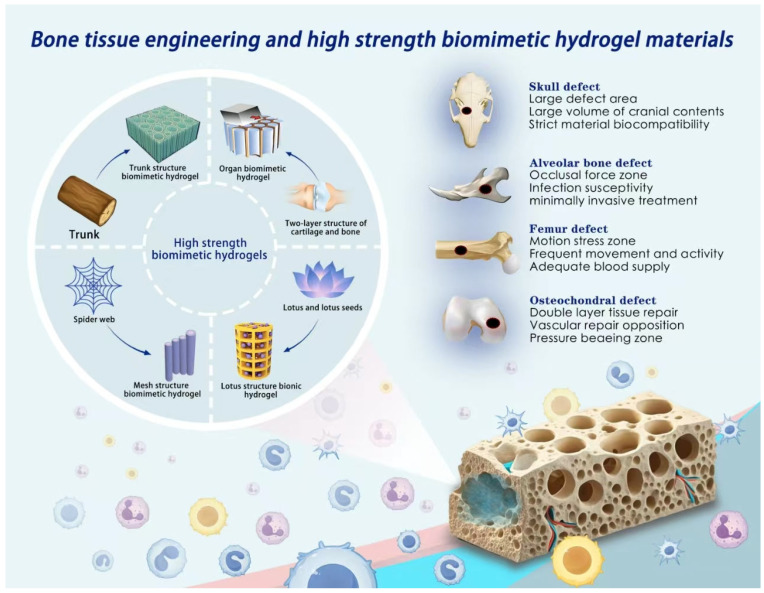
The design of hydrogel structures is inspired by natural biological tissues and natural structures: the mechanical support and stability of tree trunks, the multi-functional structure of complex organs, the ordered structure of spider webs’ porous networks, and the hierarchical porous system structure of lotus flowers. The challenges in repairing the four typical types of bone defects, the characteristics to be considered for the repair materials mainly include load-bearing capacity, mobility, tissue compatibility, and the differences in repair at different tissue interfaces.

Biomimetic strategies encompass structural biomimicry, functional biomimicry, and compositional biomimicry. Biomimetic materials are artificially engineered to emulate the operating modes and structural principles of biological systems, and include hydrogels, mineralized materials, bioceramics, and biopolymers [182]. These materials, alone or in composite forms, exhibit excellent mechanical performance and bioactivity, effectively overcoming the limitations of conventional materials. Porous plant organs often display self-healing capacity and high toughness (Figure 2). For instance, the ordered internal structure and high tensile strength of lotus fibers inspired Guan et al. to develop bacterial cellulose hydrogels mimicking the lotus fiber helical structure, which demonstrated high strength, remarkable extensibility, and good biocompatibility, making them suitable for bone and soft tissue repair [183]. Inspired by the honeycomb-like porous structure of lotus flowers, Han and others developed a biomimetic bone repair scaffold. Using 3D printed porous bioceramics as the “shell” and injecting injectable hydrogel microspheres loaded with DFO-liposomes as the “lotus seeds” inside, a hierarchical structure with built-in vascularization features was formed. Compared with traditional homogeneous hydrogels, this design achieves “structural-function integration”, that is, the ceramic scaffold provides mechanical support and osteogenic microenvironment, while the hydrogel microspheres are responsible for inducing blood vessels. Additionally, through controlled release of DFO by the microspheres, a continuous growth factor gradient is established locally to achieve precise release. Finally, the macro-micropores synergistically promote cell migration, material exchange, and vascular network extension. This biomimetic arrangement breaks through the limitations of traditional hydrogels’ single function and insufficient mechanical support. Through the coupled vascularization-osteogenesis dual-function coupling in space and time, it provides a new strategy for complex bone defect repair [184]. Wood exhibits multiscale, anisotropic, and porous structures, which have been used as templates for mineralized hydrogel composites [185]. Wang et al. prepared anisotropic hydrogel composites by in situ mineralization of hydroxyapatite (HAp) on delignified wood templates, resulting in materials with ultra-high strength and stiffness that significantly promoted osteogenic differentiation and bone integration. Due to strong intermolecular bonding and HAp reinforcement, the tensile strength of the mineralized wood hydrogel (MWH) along the longitudinal direction reached ~68 MPa with an elastic modulus of 670 MPa, surpassing most conventional high-strength hydrogels. The ordered cellulose fibrils in wood contributed to pronounced anisotropy in structure and mechanics (σ_L/σ_R = 5.1, E_L/E_R = 90.5) [186]. Chen et al. further combined oriented cellulose scaffolds with drug-loaded hydrogels to fabricate antimicrobial hydrogel composite films with excellent mechanical performance and sustained drug release [187].

Spider silk, due to its high strength, toughness, and unique hierarchical structure, has inspired hydrogel design. Dou et al. self-assembled polyacrylic acid hydrogel fibers based on spider silk structures, exhibiting outstanding mechanical performance [188]. Wu et al. prepared environmentally resilient biomimetic hydrogel fibers through ionic crosslinking and crystalline domain regulation [189]. Yang et al. integrated hyaluronic acid and tannic acid to construct hydrogels with spider silk–like heterogeneous structures, enhancing mechanical strength while enabling rapid self-healing. Cartilage itself features a stratified structure to withstand varying mechanical loads, with the superficial layer maintaining surface smoothness, the middle layer providing support, and the deep layer offering high compressive strength [190]. Zhang et al. designed a double-gradient hydrogel with a horizontally porous rigid framework and a surface water-absorbing porous layer, achieving both lubrication and load-bearing capacity [191]. Chen et al. assembled anisotropic hydrogels with hierarchical horizontal and vertical structures to develop a bilayer-oriented hydrogel with high load-bearing capacity, low friction, and superior fatigue resistance [192].

The sustainability of biomimetic materials has become increasingly important in materials science, particularly in optimizing and replacing energy-intensive processes. For example, the freeze–thaw process used in double-network (DN) hydrogels is energy-intensive, especially when multiple freeze–thaw cycles are required to enhance the crosslinking density and improve mechanical properties. This process typically demands a large amount of energy input, resulting in significant environmental impact. In contrast, solvent-based processes (such as solvent evaporation or aqueous gelation) generally consume less energy and have a lower environmental impact, especially when repeated freeze–thaw cycles are not necessary [193].

In summary, high-strength biomimetic hydrogels combine excellent biocompatibility, bioactivity, and degradability, effectively mimicking biological structures and functions, thereby advancing bone tissue engineering. Future biomimetic designs should increasingly focus on functional simulation, integrating materials science, biology, and biomimetics to promote the innovation and clinical translation of high-performance biomimetic materials.

## 6. Further Research

In the future of bone tissue engineering, the research on high-strength hydrogels needs to be deeply explored from three dimensions: clinical transformation strategies, integration of preparation methods, and sustainability throughout the entire life cycle. Firstly, systematic clinical translation research on various hydrogels should be carried out. Currently, most of these systems are still at the laboratory stage, and their long-term biological safety has not been fully verified. At the same time, complex functional hydrogels lack standardized preparation processes, which limits their batch production. In the future, a cross-disciplinary preclinical platform should be established to conduct multi-center long-term animal experiments, with a focus on clinical translation and the development of finished hydrogel products.

Secondly, the issue of uneven ion release in the body of hydrogels often remains unsolved, and a collaborative application strategy of sol–gel method and cross-linking method needs to be developed. A single method is often unable to balance injectability, mechanical strength, and stable controllability. Although the sol–gel method can achieve mild molding and uniform loading of active ingredients, the resulting gel network has low strength; chemical cross-linking can enhance mechanical properties, but it may introduce biocompatibility risks. In the future, a time-sequenced composite strategy should be focused on, such as first constructing a biomimetic primary network through sol–gel and loading growth factors, and then using light cross-linking or enzymatic cross-linking methods to achieve local strengthening, forming a system with a partitioned structure that is both rigid and flexible. This process needs to focus on solving the compatibility problem of different cross-linking mechanisms in the reaction, and using computational simulation methods to optimize process parameters, ultimately achieving programmable and precise control of the network structure.

Finally, attention must be paid to the development cost and environmental-friendly characteristics of hydrogels. Currently, high-performance hydrogels often rely on expensive raw materials and complex processes, which restrict their wide application. At the same time, issues such as the use of organic solvents during the synthesis process, the residue of non-degradable components, and the environmental impact after disposal have not been given sufficient attention. In the future, we should start from the design of sustainable materials, and establish a full life cycle evaluation system covering raw material acquisition, synthesis processing, sterilization packaging, and the environmental destination of degradation products. In conclusion, future research on high-strength hydrogels should go beyond the simplistic mindset of solely pursuing material properties. Instead, it should achieve a coordinated balance among clinical applicability, innovative preparation strategies, and system sustainability, thereby truly facilitating the transition of bone repair materials from laboratory innovation to clinical value realization.

## 7. Conclusions

High-strength hydrogels, due to their excellent biocompatibility, adjustable mechanical properties, and ability to create biomimetic microenvironments, show great promise in the field of bone tissue engineering repair. In cranial bone repair, hydrogels can closely fit the irregular defect edges, providing stable mechanical support and promoting bone integration; in load-bearing bone repairs, their biomimetic scaffold structures can guide the directional growth of new bone tissue and effectively restore the biomechanical function of the skeleton. Additionally, for alveolar bone defects, hydrogels not only promote periodontal tissue regeneration but also enhance the long-term stability of implants. In articular cartilage repair, high-strength hydrogels, due to their excellent lubrication and compressive resistance, can mimic the shock-absorbing and buffering functions of natural cartilage, significantly reducing joint friction and wear. With the interdisciplinary integration of materials science, bioengineering, and medicine, the mechanical properties, bioactivity, and long-term stability of high-strength hydrogels will continue to improve, accelerating their clinical translation process and providing more effective solutions for bone defect repair and the treatment of degenerative bone diseases. Future research should focus on improving the long-term safety of material implants, promoting the synergistic effects of angiogenesis and bone regeneration, and exploring personalized treatment strategies to better meet clinical needs.

## Figures and Tables

**Table 1:** Comparison of Mechanical Properties Between Conventional Hydrogels and Enhanced-Performance Hydrogels.

Mechanical Property	Conventional Hydrogels	Enhanced-Performance Hydrogels
Water Content	High (typically 70–99%)	High (generally maintains high water content, while mechanical strength is significantly improved)
Mechanical Strength	Relatively low, prone to brittle fracture; tensile strength typically in the range of 10–100 kPa	High strength; tensile strength can reach the MPa level, with greatly improved toughness
Toughness	Poor, characterized by brittle fracture	Excellent, exhibiting high extensibility and energy absorption capability
Elastic Recovery	Poor; slow or irreversible recovery after deformation	Good; some hydrogels can rapidly recover their original shape
Crosslinking Type	Primarily chemical crosslinking (permanent crosslinks)	Often employs multiple strategies such as double-network structures, dynamic crosslinking, nanocomposites, and sliding crosslinks
Fracture Mechanism	Fractures immediately under stress; no self-healing after fracture	Fracture is mitigated through energy dissipation mechanisms (e.g., sacrificial network rupture, dynamic bond dissociation)
Structural Complexity	Single-network structure	Complex structures including multi-network systems, nanofiller reinforcement, and dynamic crosslinking

**Table 2:** The Application of Hydrogels with Excellent Mechanical Properties in Bone Tissue Engineering.

Defects	Animals/Cells	Properties	Enhancement Methods	Results	Reference
Cranium	Rats	High mechanical properties Antibacterial property	The introduction of nanometer (CNC/TA@AgNPs)	Acceleration of bone regeneration Antimicrobial activity	[142]
	Rats	High mechanical properties Photoconductivity	The introduction of nanometer (BP@Mg)	Acceleration of bone regeneration Antimicrobial activity	[143]
	Rats	High mechanical properties Low expandability	The introduction of nanometer (Ag/BC@HAp)	Antimicrobial activity	[144]
	Rats	Injectability High mechanical properties	The introduction of nanometer (MSN)	Bone regeneration Closure wound	[147]
	Rats	High mechanical properties	The introduction of inorganics (POSS)	Angiogenesis Bone regeneration	[148]
Femur	Rats	High mechanical properties	The introduction of nanometer (MgO)	Angiogenesis Bone regeneration	[152]
	Normal human osteoblasts	Photoreactivity Injectable Biocompatibility High mechanical properties	The introduction of nanometer (GO)	Osteoinductivity Bone reconstruction	[153]
	Rats	Antibacterial Osteogenic Anti-inflammatory	Ion(Fe/Sr) replacement of HAP	Acceleration of osseointegration Antimicrobial activity	[154]
	Rabbits	Self-healing Injectability Biocompatibility	Dynamic crosslinking	Bone regeneration Reduced inflammation	[155]
	Rats	High mechanical properties Injectability	Introduction of crosslinking agents (OCMC and CMCS)	Acceleration of bone reconstruction	[156]
	Rats	High mechanical properties	Introduction of nanometer (BPNSs)	Osteogenesis	[157]
	Rats	High mechanical properties High tenacity	Enzyme mineralization	Osteogenesis	[158]
Alveolar bone	Rats	High mechanical properties	Fabrication of support structure (PLAG)	Osteogenesis	[159]
	Rats	High mechanical properties Antibacterial property	Introduction of nanometer (CNP)	Osteogenesis Antibacterial property	[164]
	Rats	High mechanical properties Anti-inflammatory property	Introduction of TA	Antibacterial property Osteogenesis	[165]
	Rats	Injectability Anti-inflammatory property Osteoconductivity	Electrostatic interactions Schiff base formation	Periodontal tissue regeneration	[166]
	Rats	Injectability Antibacterial property High mechanical properties	π-π interactions Photo-crosslinking	Antibacterial property Osteogenesis	[167]
	Rats	Injectability High mechanical properties	Introduction of nanometer (MXene)	Alveolar bone regeneration	[168]
	Rats	Antibacterial property	Introduction of inorganics (nHA)	Anti-inflammatory Antibacterial property Periodontal tissue regeneration	[169]
	Franz cells	High mechanical properties Injectability	Bilayered thin film	Osteogenesis	[170]
	Rats	High mechanical properties Antibacterial property	Sol-gel transition	Antibacterial property Persistent drug release	[172]
Arthrosis	Rats	High mechanical properties Injectability	Formation of cross-linked networks	Provision of immune microenvironment	[173]
	Rabbits	High mechanical properties	3d bioprinting technology (PBP)	Promotion of osteoblast differentiation	[176]
	Rabbits	High mechanical properties Biocompatibility Conductivity	Introduction of EGCG	Efficient Cartilage and Subchondral Bone Regeneration	[177]
	Rats	Biocompatibility Degradable High mechanical properties Injectability	Formation of double network hydrogels (OSAGC)	Cartilage and Subchondral Bone Regeneration Subchondral Bone Regeneration	[178]
	Rats	Injectability High mechanical Properties	C bond formation	Osteochondral regeneration Repair of osteochondral defects	[179]
	Rats	Injectability High mechanical Properties	‘Building block’ properties	Osteochondral regeneration	[180]

## Data Availability

No new data were created or analyzed in this study.

## Abbreviations

3D	Three-dimensional
OA	Osteoarthritis
MSC	Mesenchymal Stem Cell
PCL	Polycaprolactone
PRP	Platelet-Rich Plasma
GelMA	Gelatin Methacryloyl
FEK	Phenylalanine–Glutamic acid–Lysine octapeptide
OSAGC	Oxidized Sodium Alginate/Gelatin/Chondroitin Sulfate
CS	Chitosan
HAp	Hydroxyapatite
ABG	Bioactive Glass
ABGNs	Amino-Functionalized Bioactive Glass Nanoparticles
DN	Double Network
TN	Triple Network
BG	Bioactive Glass
BGNs	Bioactive Glass Nanoparticles
PLGA	Poly(lactic-co-glycolic acid)
MgO	Magnesium Oxide
GO	Graphene Oxide
PVA	Polyvinyl Alcohol
CMC	Carboxymethyl Cellulose
EMF	Electromagnetic Field
SCAP	Stem Cells from the Apical Papilla
PNIPAM	Poly(N-isopropylacrylamide)
HAPAM	Hydroxypropyl Acrylamide
BSA	Bovine Serum Albumin
PPy	Polypyrrole
P(AM-co-AA)	Poly(Acrylamide-co-Acrylic Acid)
σ_L/σ_R	Ratio of longitudinal to radial tensile strength
E_L/E_R	Ratio of longitudinal to radial elastic modulus
MWH	Mineralized Wood Hydrogel
PAA	Polyacrylic Acid
HA	Hyaluronic Acid
TA	Tannic Acid
PIEZO1	Piezo Type Mechanosensitive Ion Channel Component 1
ITGα5	Integrin Alpha 5
DMD	Digital Micromirror Device
LED	Light-Emitting Diode
PL	Peptide/Peptide Linker
nHA	Nano-Hydroxyapatite
β-GP	Beta-Glycerophosphate
EGCG	Epigallocatechin Gallate
CM	Carboxymethyl

## References

[1] Wang J., Wu Y., Li G., Zhou F., Wu X., Wang M., Liu X., Tang H., Bai L., Geng Z. (2024). Engineering Large-Scale Self-Mineralizing Bone Organoids with Bone Matrix-Inspired Hydroxyapatite Hybrid Bioinks. Adv. Mater..

[2] Sivakumar P.M., Yetisgin A.A., Sahin S.B., Demir E., Cetinel S. (2022). Bone tissue engineering: Anionic polysaccharides as promising scaffolds. Carbohydr. Polym..

[3] Sun T., Li Q., Cheng H., Zhang W., Han N., Kou Y. (2025). Bone Tissue Engineering Scaffolds for Bone Aging: Drug Delivery and Microenvironment Regulation. Tissue Eng. Part B Rev..

[4] Santoro A., Voto A., Fortino L., Guida R., Laudisio C., Cillo M., D’Ursi A.M. (2025). Bone defect treatment in regenerative medicine: Exploring natural and synthetic bone substitutes. Int. J. Mol. Sci..

[5] Moriarty T.F., Metsemakers W.J., Morgenstern M., Hofstee M.I., Vallejo Diaz A., Cassat J.E., Wildemann B., Depypere M., Schwarz E.M., Richards R.G. (2022). Fracture-related infection. Nat. Rev. Dis. Primers.

[6] Wu Z., Shi G., Li L., Piao Z., Wang J., Chen R., Hao Z., Zhang Z., Li Z., Huang Y. (2025). Recent advances in smart responsive hydrogel microspheres for tissue regeneration: Preparation, characteristics and applications. Mater. Horiz..

[7] Aytac Z., Dubey N., Daghrery A., Ferreira J.A., de Souza Araújo I.J., Castilho M., Malda J., Bottino M.C. (2022). Innovations in Craniofacial Bone and Periodontal Tissue Engineering—From Electrospinning to Converged Biofabrication. Int. Mater. Rev..

[8] Jia B., Zhao X., Wan X., Wu Z., Wu Y., Huang H. (2025). Biofunctional and Interface-Engineered Hydrogels for Advanced Tissue Engineering. Adv. Healthc. Mater..

[9] Masters E.A., Ricciardi B.F., Bentley K.L.M., Moriarty T.F., Schwarz E.M., Muthukrishnan G. (2022). Skeletal infections: Microbial pathogenesis, immunity and clinical management. Nat. Rev. Microbiol..

[10] Khattak S., Ullah I., Sohail M., Akbar M.U., Rauf M.A., Ullah S., Shen J., Xu H.T. (2025). Endogenous/exogenous stimuli-responsive smart hydrogels for diabetic wound healing. Aggregate.

[11] Yuan X., Zhu W., Yang Z., He N., Chen F., Han X., Zhou K. (2024). Recent Advances in 3D Printing of Smart Scaffolds for Bone Tissue Engineering and Regeneration. Adv. Mater..

[12] Zheng M., Guo J., Li Q., Yang J., Han Y., Yang H., Yu M., Zhong L., Lu D., Li L. (2021). Syntheses and characterization of anti-thrombotic and anti-oxidative Gastrodin-modified polyurethane for vascular tissue engineering. Bioact. Mater..

[13] Kamaraj M., Moghimi N., McCarthy A., Chen J., Cao S., Chethikkattuveli Salih A.R., Joshi A., Jucaud V., Panayi A., Shin S.R. (2024). Granular Porous Nanofibrous Microspheres Enhance Cellular Infiltration for Diabetic Wound Healing. ACS Nano.

[14] Xu C., Su P., Chen X., Meng Y., Yu W., Xiang A.P., Wang Y. (2011). Biocompatibility and osteogenesis of biomimetic Bioglass-Collagen-Phosphatidylserine composite scaffolds for bone tissue engineering. Biomaterials.

[15] Vallet-Regí M., Colilla M., González B. (2011). Medical applications of organic-inorganic hybrid materials within the field of silica-based bioceramics. Chem. Soc. Rev..

[16] Li Z., Yang T., Li X., Yin P., Yang B., Li D., Wang Y., Teng W., Yu Q., Li W. (2025). A Bio-Responsive Hydrogel with Spatially Heterogeneous Structure for Treating Infectious Tissue Injuries. Adv. Sci..

[17] Wang Z., Zhang Y., Yin Y., Liu J., Li P., Zhao Y., Bai D., Zhao H., Han X., Chen Q. (2022). High-Strength and Injectable Supramolecular Hydrogel Self-Assembled by Monomeric Nucleoside for Tooth-Extraction Wound Healing. Adv. Mater..

[18] Cui S., Zhang S., Coseri S. (2023). An Injectable and Self-Healing Cellulose Nanofiber-Reinforced Alginate Hydrogel for Bone Repair. Carbohydr. Polym..

[19] Zhang Y., Song Q., Yang S., Xiao J., Wang F., Fu C., Xing X., Wu J., Zhang S., Zhu Y. (2025). Revitalizing Osteoporotic Bone Repair via Multilevel ROS Scavenging and Osteoimmune Regulating Hydrogel. Compos. Part B Eng..

[20] Li Y., Wang Y., Ding Y., Fan X., Ye L., Pan Q., Zhang B., Li P., Luo K., Hu B. (2024). A Double Network Composite Hydrogel with Self-Regulating Cu^2+^/Luteolin Release and Mechanical Modulation for Enhanced Wound Healing. ACS Nano.

[21] He G., Xian Y., Lin H., Yu C., Chen L., Chen Z., Hong Y., Zhang C., Wu D. (2024). An Injectable and Coagulation-Independent Tetra-PEG Hydrogel Bioadhesive for Post-Extraction Hemostasis and Alveolar Bone Regeneration. Bioact. Mater..

[22] Zhao C., Zhou L., Chiao M., Yang W. (2020). Antibacterial Hydrogel Coating: Strategies in Surface Chemistry. Adv. Colloid Interface Sci..

[23] Sha Z., Chen X., Song H., Zheng Y., Cui W., Ran R. (2025). Toughening Hydrogels with Small Molecules: Tiny Matter, Big Impact. Mater. Horiz..

[24] Ajdary R., Tardy B.L., Mattos B.D., Bai L., Rojas O.J. (2021). Plant Nanomaterials and Inspiration from Nature: Water Interactions and Hierarchically Structured Hydrogels. Adv. Mater..

[25] Wang S., Chi J., Jiang Z., Hu H., Yang C., Liu W., Han B. (2021). A Self-Healing and Injectable Hydrogel Based on Water-Soluble Chitosan and Hyaluronic Acid for Vitreous Substitute. Carbohydr. Polym..

[26] He Y., Zhang Y., Xiang B., Fu C., Zhang H., Zhan D., Guo Z. (2025). Extremely Fast Wicking Self-Layered Interpenetrating Network Hydrogel for Rapid All Day Collection of Atmospheric Water. Adv. Funct. Mater..

[27] Ren Z., Guo F., Wen Y., Yang Y., Liu J., Cheng S. (2024). Strong and Anti-Swelling Nanofibrous Hydrogel Composites Inspired by Biological Tissue for Amphibious Motion Sensors. Mater. Horiz..

[28] Hu M., Zhang Q., Qin L. (2025). Innovative Applications of Multidimensional Engineered Hydrogels in Wound Healing. J. Adv. Res..

[29] Zhu M., Zhang H., Zhou Q., Sheng S., Gao Q., Geng Z., Chen X., Lai Y., Jing Y., Xu K. (2025). Dynamic GelMA/DNA Dual-Network Hydrogels Promote Woven Bone Organoid Formation and Enhance Bone Regeneration. Adv. Mater..

[30] Na H., Kang Y.W., Park C.S., Jung S., Kim H.Y., Sun J.Y. (2022). Hydrogel-based Strong and Fast Actuators by Electroosmotic Turgor Pressure. Science.

[31] Meng X., Qiao Y., Do C., Bras W., He C., Ke Y., Russell T.P., Qiu D. (2022). Hysteresis-Free Nanoparticle-Reinforced Hydrogels. Adv. Mater..

[32] Vernerey F.J., Lalitha Sridhar S., Muralidharan A., Bryant S.J. (2021). Mechanics of 3D Cell-Hydrogel Interactions: Experiments, Models, and Mechanisms. Chem. Rev..

[33] Chen Z., Ezzo M., Zondag B., Rakhshani F., Ma Y., Hinz B., Kumacheva E. (2024). Intrafibrillar Crosslinking Enables Decoupling of Mechanical Properties and Structure of a Composite Fibrous Hydrogel. Adv. Mater..

[34] Zou J., Lin Z., Zhan L., Qin Y., Sun Q., Ji N., Xie F. (2024). A Short Linear Glucan Nanocomposite Hydrogel Formed by In Situ Self-Assembly with Highly Elastic, Fatigue-Resistant and Self-Recovery. Carbohydr. Polym..

[35] Zhao Y., Cui J., Qiu X., Yan Y., Zhang Z., Fang K., Yang Y., Zhang X., Huang J. (2022). Manufacturing and Post-Engineering Strategies of Hydrogel Actuators and Sensors: From Materials to Interfaces. Adv. Colloid Interface Sci..

[36] Shao G., Wang S., Zhao H., Zhao G., Yang L., Zhu L., Liu H. (2022). Tunable Arrangement of Hydrogel and Cyclodextrin-based Metal Organic Frameworks Suitable for Drug Encapsulation and Release. Carbohydr. Polym..

[37] Wang J., Lu T., Li Y., Wang J., Spruijt E. (2023). Aqueous Coordination Polymer Complexes: From Colloidal Assemblies to Bulk Materials. Adv. Colloid Interface Sci..

[38] Dhand A.P., Galarraga J.H., Burdick J.A. (2021). Enhancing Biopolymer Hydrogel Functionality through Interpenetrating Networks. Trends Biotechnol..

[39] Wu T., Cui C., Fan C., Xu Z., Liu Y., Liu W. (2021). Tea Eggs-inspired High-strength Natural Polymer Hydrogels. Bioact. Mater..

[40] Sun M., Ji G., Li M., Zheng J. (2025). Molecularly Engineered Hydrogel Electrolyte Embedded with Multifunctional Oxygen-Rich Macrocyclic Units for Uniform Zinc Deposition. Adv. Sci..

[41] Zhang D., Chen H., Zhang Y., Yang J., Chen Q., Wu J., Liu Y., Zhao C., Tang Y., Zheng J. (2025). Antifreezing Hydrogels: From Mechanisms and Strategies to Applications. Chem. Soc. Rev..

[42] Wang H., Wei Z., Liu Z., Zheng B., Zhang Z., Yan X., He L., Li T., Zhao D. (2025). Energy Dissipation and Toughening of Covalent Networks via a Sacrificial Conformation Approach. Angew. Chem. Int. Ed..

[43] Yang Y., Hu Q., Shao Q., Peng Y., Yu B., Luo F., Chen J., Xu C., Li Z., Tam M. (2025). A Baicalin-Based Functional Polymer in Dynamic Reversible Networks Alleviates Osteoarthritis by Cellular Interactions. Adv. Sci..

[44] Nie J., Fu J., He Y. (2020). Hydrogels: The Next Generation Body Materials for Microfluidic Chips?. Small.

[45] Dhand A.P., Davidson M.D., Galarraga J.H., Qazi T.H., Locke R.C., Mauck R.L., Burdick J.A. (2022). Simultaneous One-Pot Interpenetrating Network Formation to Expand 3D Processing Capabilities. Adv. Mater..

[46] Wang F., Han X., Han Z., Wang J., Cai Z., Chen G., Bai D., Cui W. (2025). Slide-Ring Structured Stress-Electric Coupling Hydrogel Microspheres for Low-Loss Transduction Between Tissues. Adv. Mater..

[47] Shen J., Chen A., Cai Z., Chen Z., Cao R., Liu Z., Li Y., Hao J. (2022). Exhausted Local Lactate Accumulation via Injectable Nanozyme-Functionalized Hydrogel Microsphere for Inflammation Relief and Tissue Regeneration. Bioact. Mater..

[48] Yang Q., Miao Y., Luo J., Chen Y., Wang Y. (2023). Amyloid Fibril and Clay Nanosheet Dual-Nanoengineered DNA Dynamic Hydrogel for Vascularized Bone Regeneration. ACS Nano.

[49] Yun J., Woo H.T., Lee S., Cha H.J. (2025). Visible Light-Induced Simultaneous Bioactive Amorphous Calcium Phosphate Mineralization and in situ Crosslinking of Coacervate-Based Injectable Underwater Adhesive Hydrogels for Enhanced Bone Regeneration. Biomaterials.

[50] Liu J., Li W., Yu S., Blanchard S., Lin S. (2024). Fatigue-Resistant Mechanoresponsive Color-Changing Hydrogels for Vision-Based Tactile Robots. Adv. Mater..

[51] Jiang Z., Feng J., Wang F., Wang J., Wang N., Zhang M., Hsieh C.Y., Hou T., Cui W., Ma L. (2025). AI-Guided Design of Antimicrobial Peptide Hydrogels for Precise Treatment of Drug-Resistant Bacterial Infections. Adv. Mater..

[52] Bertsch P., Diba M., Mooney D.J., Leeuwenburgh S.C.G. (2023). Self-Healing Injectable Hydrogels for Tissue Regeneration. Chem. Rev..

[53] Liu Y., Yang X., Miao Y., Chen T., Gao W., Zhou G., Jia G., Yang X., Zhang J., Jin Y. (2026). Self-Supported DNA Hydrogel Facilitates Microenvironment Remodeling and Cartilage Repair to Prevent Osteoarthritis Progression via an Ambidextrous Strategy. Biomaterials.

[54] Zhou H., He Z., Cao Y., Chu L., Liang B., Yu K., Deng Z. (2024). An Injectable Magnesium-Loaded Hydrogel Releases Hydrogen to Promote Osteoporotic Bone Repair via ROS Scavenging and Immunomodulation. Theranostics.

[55] Berradi A., Lafdali A., Ouazzani N., Aziz K., Mandi L., El Achaby M., Kurniawan T.A., Aziz F. (2025). Development and Characterization of a Carboxymethyl Cellulose-Alginate Hybrid Superabsorbent Hydrogel Designed for Water Management in Agriculture. Int. J. Biol. Macromol..

[56] Zhao X., Luo J., Huang Y., Mu L., Chen J., Liang Z., Yin Z., Chu D., Han Y., Guo B. (2023). Injectable Antiswelling and High-Strength Bioactive Hydrogels with a Wet Adhesion and Rapid Gelling Process to Promote Sutureless Wound Closure and Scar-free Repair of Infectious Wounds. ACS Nano.

[57] Qian Y., Zheng Y., Jin J., Wu X., Xu K., Dai M., Niu Q., Zheng H., He X., Shen J. (2022). Immunoregulation in Diabetic Wound Repair with a Photoenhanced Glycyrrhizic Acid Hydrogel Scaffold. Adv. Mater..

[58] Li S., Chandra Biswas M., Ford E. (2022). Dual Roles of Sodium Polyacrylate in Alginate Fiber Wet-Spinning: Modify the Solution Rheology and Strengthen the Fiber. Carbohydr. Polym..

[59] Wang Y., Zhao P.C., Sun J., Liang J., Shen T., Li C.H., Jin Z. (2025). Titanium-Polyoxometalate Crosslinked Metallosupramolecular Polymer as Artificial Interfacial Layer for Highly Persistent and Low-Temperature Tolerant Lithium Metal Batteries. Angew. Chem. Int. Ed..

[60] Sattari S., Mariano C.A., Eskandari M. (2023). Biaxial Mechanical Properties of the Bronchial Tree: Characterization of Elasticity, Extensibility, and Energetics, Including the Effect of Strain Rate and Preconditioning. Acta Biomater..

[61] Hu X., Mei S., Wang F., Tang S., Xie D., Ding C., Du W., Zhao J., Yang L., Wu Z. (2021). A Microporous Surface Containing Si₃N₄/Ta Microparticles of PEKK Exhibits Both Antibacterial and Osteogenic Activity for Inducing Cellular Response and Improving Osseointegration. Bioact. Mater..

[62] Wang Q., Chen Y., Ding H., Cai Y., Yuan X., Lv J., Huang J., Huang J., Zhang C., Hong Z. (2025). Optogenetic Activation of Mechanical Nociceptions to Enhance Implant Osseointegration. Nat. Commun..

[63] Hwang J.H., Kim E., Lim E.Y., Lee W., Kim J.O., Choi I., Kim Y.S., Kim D.G., Lee J.H., Lee J.C. (2023). A Multifunctional Interlocked Binder with Synergistic In Situ Covalent and Hydrogen Bonding for High-Performance Si Anode in Li-ion Batteries. Adv. Sci..

[64] Cao H., Duan L., Zhang Y., Cao J., Zhang K. (2021). Current Hydrogel Advances in Physicochemical and Biological Response-Driven Biomedical Application Diversity. Signal Transduct. Target. Ther..

[65] Liu X., Inda M.E., Lai Y., Lu T.K., Zhao X. (2022). Engineered Living Hydrogels. Adv. Mater..

[66] Li M., Tian J., Yu K., Liu H., Yu X., Wang N., Gong Q., Li K., Shen Y., Wei X. (2024). A ROS-Responsive Hydrogel Incorporated with Dental Follicle Stem Cell-Derived Small Extracellular Vesicles Promotes Dental Pulp Repair by Ameliorating Oxidative Stress. Bioact. Mater..

[67] Zhang W., Liu S., Wang L., Li B., Xie M., Deng Y., Zhang J., Zeng H., Qiu L., Huang L. (2024). Triple-Crosslinked Double-Network Alginate/Dextran/Dendrimer Hydrogel with Tunable Mechanical and Adhesive Properties: A Potential Candidate for Sutureless Keratoplasty. Carbohydr. Polym..

[68] Zhang J., Chen L., Chen L., Qian S., Mou X., Feng J. (2021). Highly Antifouling, Biocompatible and Tough Double Network Hydrogel Based on Carboxybetaine-Type Zwitterionic Polymer and Alginate. Carbohydr. Polym..

[69] Zhu J., Xie F., Qiu Z., Chen L. (2024). Effect of Active Carbonyl-Carboxyl Ratio on Dynamic Schiff Base Crosslinking and Its Modulation of High-Performance Oxidized Starch-Chitosan Hydrogel by Hot Extrusion 3D Printing. Carbohydr. Polym..

[70] Yang Y., He G., Pan Z., Zhang K., Xian Y., Zhu Z., Hong Y., Zhang C., Wu D. (2024). An Injectable Hydrogel with Ultrahigh Burst Pressure and Innate Antibacterial Activity for Emergency Hemostasis and Wound Repair. Adv. Mater..

[71] Zhang H., Gan X., Yan Y., Zhou J. (2024). A Sustainable Dual Cross-Linked Cellulose Hydrogel Electrolyte for High-Performance Zinc-Metal Batteries. Nanomicro Lett..

[72] Xiao K., Wang Z., Wu Y., Lin W., He Y., Zhan J., Luo F., Li Z., Li J., Tan H. (2019). Biodegradable, Anti-Adhesive and Tough Polyurethane Hydrogels Crosslinked by Triol Crosslinkers. J. Biomed. Mater. Res. A.

[73] Li Q., Quan X., Xu S., Hu Z., Hu R., Li G., Han B., Ji X. (2025). Multifunctional Network-Shaped Hydrogel Assemblies. Small.

[74] Tong R., Ma Z., Gu P., Yao R., Li T., Zeng M., Guo F., Liu L., Xu J. (2023). Stretchable and Sensitive Sodium Alginate Ionic Hydrogel Fibers for Flexible Strain Sensors. Int. J. Biol. Macromol..

[75] Wu L., Kang Y., Shi X., Yuezhen B., Qu M., Li J., Wu Z.S. (2023). Natural-Wood-Inspired Ultrastrong Anisotropic Hybrid Hydrogels Targeting Artificial Tendons or Ligaments. ACS Nano.

[76] Khanmohammadi A., Sadighian S., Ramazani A. (2022). Anti-plasmodial Effects of Quinine-Loaded Magnetic Nanocomposite Coated with Heparin. Int. J. Pharm..

[77] Lv M., Sun D.W., Huang L., Pu H. (2022). Precision Release Systems of Food Bioactive Compounds Based on Metal-Organic Frameworks: Synthesis, Mechanisms and Recent Applications. Crit. Rev. Food Sci. Nutr..

[78] Ding Y., Ma R., Liu G., Li X., Xu K., Liu P., Cai K. (2023). Fabrication of a New Hyaluronic Acid/Gelatin Nanocomposite Hydrogel Coating on Titanium-Based Implants for Treating Biofilm Infection and Excessive Inflammatory Response. ACS Appl. Mater. Interfaces.

[79] Chen F., Qin J., Wu P., Gao W., Sun G. (2023). Glucose-Responsive Antioxidant Hydrogel Accelerates Diabetic Wound Healing. Adv. Healthc. Mater..

[80] Cai J., Luo W., Pan J., Li G., Pu Y., Si L., Shi G., Shao Y., Ma H., Guan J. (2022). Glucose-Sensing Photonic Nanochain Probes with Color Change in Seconds. Adv. Sci..

[81] Xiao L., Xie P., Ma J., Shi K., Dai Y., Pang M., Luo J., Tan Z., Ma Y., Wang X. (2023). A Bioinspired Injectable, Adhesive, and Self-Healing Hydrogel with Dual Hybrid Network for Neural Regeneration after Spinal Cord Injury. Adv. Mater..

[82] Taylor D.L., In Het Panhuis M. (2016). Self-Healing Hydrogels. Adv. Mater..

[83] Zou L., Li Y., Feng S., Wang Z., Xiao H., Chen S., Wang Y., He L., Mao X. (2025). Innovations and Applications of Composite Hydrogels: From Polymer-Based Systems to Metal-Ion-Doped and Functional Nanomaterial-Enhanced Architectures. Small.

[84] Song Y., Chen X., Dabade V., Shield T.W., James R.D. (2013). Enhanced Reversibility and Unusual Microstructure of a Phase-Transforming Material. Nature.

[85] Kloxin C.J., Bowman C.N. (2013). Covalent Adaptable Networks: Smart, Reconfigurable and Responsive Network Systems. Chem. Soc. Rev..

[86] Awad W.M., Davies D.W., Kitagawa D., Halabi J.M., Al-Handawi M.B., Tahir I., Tong F., Campillo-Alvarado G., Shtukenberg A.G., Alkhidir T. (2023). Mechanical Properties and Peculiarities of Molecular Crystals. Chem. Soc. Rev..

[87] Ge S., Li J., Geng J., Liu S., Xu H., Gu Z. (2021). Adjustable Dual Temperature-Sensitive Hydrogel Based on a Self-Assembly Cross-Linking Strategy with Highly Stretchable and Healable Properties. Mater. Horiz..

[88] Wang Y., Chen P., Ding Y., Zhu P., Liu Y., Wang C., Gao C. (2024). Multifunctional Nano-Conductive Hydrogels with High Mechanical Strength, Toughness and Fatigue Resistance as Self-Powered Wearable Sensors and Deep Learning-Assisted Recognition System. Adv. Funct. Mater..

[89] Zhang Y., Chen K., Li Y., Lan J., Yan B., Shi L., Ran R. (2019). High-Strength, Self-Healable, Temperature-Sensitive, MXene-Containing Composite Hydrogel as a Smart Compression Sensor. ACS Appl. Mater. Interfaces.

[90] Cai S., Niu B., Ma X., Wan S., He X. (2022). High Strength, Recyclable, Anti-Swelling and Shape-Memory Hydrogels Based on Crystal Microphase Crosslinking and Their Application as Flexible Sensor. Chem. Eng. J..

[91] Yang Y., Chen S., Niu L., Wang R. (2024). Microgel-Crosslinked Thermo-Responsive Hydrogel Actuators with High Mechanical Properties and Rapid Response. Macromol. Rapid Commun..

[92] Liu D., Huyan C., Wang Z., Guo Z., Zhang X., Torun H., Mulvihill D., Xu B.B., Chen F. (2023). Conductive Polymer Based Hydrogels and Their Application in Wearable Sensors: A Review. Mater. Horiz..

[93] Wang T., Liu J., Zhao Y., Lu Y. (2025). Synergistic Mastery: Advancing Mechanical and Electrical Harmony in Conducting Polymer Hydrogel Bioelectronics. Bioact. Mater..

[94] Zhu T., Ni Y., Biesold G.M., Cheng Y., Ge M., Li H., Huang J., Lin Z., Lai Y. (2023). Recent Advances in Conductive Hydrogels: Classifications, Properties, and Applications. Chem. Soc. Rev..

[95] Guo Y., Bae J., Fang Z., Li P., Zhao F., Yu G. (2020). Hydrogels and Hydrogel-Derived Materials for Energy and Water Sustainability. Chem. Rev..

[96] Yang J., Xu F., Han C.-R. (2017). Metal Ion Mediated Cellulose Nanofibrils Transient Network in Covalently Cross-Linked Hydrogels: Mechanistic Insight into Morphology and Dynamics. Biomacromolecules.

[97] Xu J., Zhang H., Guo Z., Zhang C., Tan H., Gong G., Yu M., Xu L. (2023). Fully Physical Crosslinked BSA-Based Conductive Hydrogels with High Strength and Fast Self-Recovery for Human Motion and Wireless Electrocardiogram Sensing. Int. J. Biol. Macromol..

[98] Kumar J., Tolepbergenova M., Musayev A., Danyshbayeva A., Begimbekova L., Kumar H., Gulnar T., Kumar P., Shamim S. (2025). Stimuli-Responsive Hydrogels for Targeted Antibiotic Delivery in Bone Tissue Engineering. AAPS PharmSciTech.

[99] Chen Z., Lv Z., Zhang Z., Weitz D.A., Zhang H., Zhang Y., Cui W. (2021). Advanced Microfluidic Devices for Fabricating Multi-Structural Hydrogel Microsphere. Wiley Online Libr..

[100] Zhang C., Zhang Z., Qi Y. (2021). Preparation, Structure, and Properties of Polystyrene-Microsphere-Reinforced PEG-Based Hydrogels. Polymers.

[101] Tolabi H., Davari N., Khajehmohammadi M., Malektaj H., Nazemi K., Vahedi S., Ghalandari B., Reis R.L., Ghorbani F., Oliveira J.M. (2023). Progress of Microfluidic Hydrogel-Based Scaffolds and Organ-on-Chips for the Cartilage Tissue Engineering. Adv. Mater..

[102] Montanari E., Meier R.P.H., Mahou R., Seebach J.D., Wandrey C., Gerber-Lemaire S., Buhler L.H., Gonelle-Gispert C. (2017). Multipotent Mesenchymal Stromal Cells Enhance Insulin Secretion from Human Islets via N-cadherin Interaction and Prolong Function of Transplanted Encapsulated Islets in Mice. Stem Cell Res. Ther..

[103] Xie F., Boyer C., Gaborit V., Rouillon T., Guicheux J., Tassin J.F., Geoffroy V., Réthoré G., Weiss P. (2018). A Cellulose/Laponite Interpenetrated Polymer Network (IPN) Hydrogel: Controllable Double-Network Structure with High Modulus. Polymers.

[104] Kim S., Kim M., Koh W.G. (2021). Preparation of Surface-Reinforced Superabsorbent Polymer Hydrogel Microspheres via Incorporation of In Situ Synthesized Silver Nanoparticles. Polymers.

[105] Yang C., Ma X., Wu P., Shang L., Zhao Y., Zhong L. (2023). Adhesive Composite Microspheres with Dual Antibacterial Strategies for Infected Wound Healing. Small.

[106] Li X., Wang L., Wang J., Fan H., Pang F., He W., Jiang C., Jin Y., Shen Y., Wang Y. (2025). Enhanced Osteoarthritis Treatment Using an Injectable pH-Responsive and Cartilage-Targeted Liposome-Anchored Kartogenin-Incorporated Methacrylated Gelatin Hydrogel Microspheres. Int. J. Biol. Macromol..

[107] Fu K. (2025). What 3D Printing Cannot Achieve: Rethinking Composite Additive Manufacturing. Acc. Mater. Res..

[108] Yang J., Wang H., Huang W., Peng K., Shi R., Tian W., Lin L., Yuan J., Yao W., Ma X. (2023). A Natural Polymer-Based Hydrogel with Shape Controllability and High Toughness and Its Application to Efficient Osteochondral Regeneration. Mater. Horiz..

[109] Puza F., Lienkamp K. (2022). 3D Printing of Polymer Hydrogels—From Basic Techniques to Programmable Actuation. Adv. Funct. Mater..

[110] Chen J., Liu X., Tian Y., Zhu W., Yan C., Shi Y., Kong L.B., Qi H.J., Zhou K. (2022). 3D-Printed Anisotropic Polymer Materials for Functional Applications. Adv. Mater..

[111] Park S., Shou W., Makatura L., Matusik W., Fu K.K. (2022). 3D Printing of Polymer Composites: Materials, Processes, and Applications. Matter.

[112] Lin X., Zhao X., Xu C., Wang L., Xia Y. (2022). Progress in the Mechanical Enhancement of Hydrogels: Fabrication Strategies and Underlying Mechanisms. J. Polym. Sci..

[113] Dong X., Liu Q., Gan S.W., Zhuo H., Li T., Zhao Y., Zhai W. (2024). A Hierarchical Hydrogel Impregnation Strategy Enables Brittle-Failure-Free 3D-Printed Bioceramic Scaffolds. Small.

[114] Zhao Q., Liu C., Chang Y., Wu H., Hou Y., Wu S., Guo M. (2023). Low-Temperature 3D Printing Technology of Poly(vinyl alcohol) Matrix Conductive Hydrogel Sensors with Diversified Path Structures and Good Electric Sensing Properties. Sensors.

[115] Dutta S.D., Bin J., Ganguly K., Patel D.K., Lim K.-T. (2021). Electromagnetic Field-Assisted Cell-Laden 3D Printed Poloxamer-407 Hydrogel for Enhanced Osteogenesis. RSC Adv..

[116] Zhang Y.S., Khademhosseini A. (2017). Advances in Engineering Hydrogels. Science.

[117] Rodin M., Li J., Kuckling D. (2021). Dually Cross-Linked Single Networks: Structures and Applications. Chem. Soc. Rev..

[118] Hasan N., Bhuyan M.M., Jeong J.-H. (2024). Single/Multi-Network Conductive Hydrogels—A Review. Polymers.

[119] Ning X., Huang J., Yimuhan A., Yuan N., Chen C., Lin D. (2022). Research Advances in Mechanical Properties and Applications of Dual Network Hydrogels. Int. J. Mol. Sci..

[120] Li L., Wu P., Yu F., Ma J. (2022). Double Network Hydrogels for Energy/Environmental Applications: Challenges and Opportunities. J. Mater. Chem. A.

[121] Guo X., Dong Y., Qin J., Zhang Q., Zhu H., Zhu S. (2025). Fracture-Resistant Stretchable Materials: An Overview from Methodology to Applications. Adv. Mater..

[122] Li X., Gong J.P. (2024). Design Principles for Strong and Tough Hydrogels. Nat. Rev. Mater..

[123] He W., Wen J., Hu Q., Yi Y., Wei Z., Yang X., Zhai G., Li F., Ye L. (2025). The Advances in Zwitterionic Materials and Their Biomedical Applications. Int. Mater. Rev..

[124] Nie L., Sun Y., Okoro O.V., Deng Y., Jiang G., Shavandi A. (2023). Click Chemistry for 3D Bioprinting. Mater. Horiz..

[125] Zhang X., Chen Q., Chen K., Feng C., Feng H., Li X., Zhang D. (2024). A Robust Low-Friction Triple Network Hydrogel Based on Multiple Synergistic Enhancement Mechanisms. Friction.

[126] Yang J., Li K., Tang C., Liu Z., Fan J., Qin G., Cui W., Zhu L., Chen Q. (2022). Recent Progress in Double Network Elastomers: One Plus One Is Greater than Two. Adv. Funct. Mater..

[127] Huang C., Yu M., Li H., Wan X., Ding Z., Zeng W., Zhou Z. (2021). Research Progress of Bioactive Glass and Its Application in Orthopedics. Adv. Mater. Interfaces.

[128] Zhang W., Liu X., Wang J., Tang J., Hu J., Lu T., Suo Z. (2018). Fatigue of Double-Network Hydrogels. Eng. Fract. Mech..

[129] Zhao F., Yang Z., Xiong H., Yan Y., Chen X., Shao L. (2023). A Bioactive Glass Functional Hydrogel Enhances Bone Augmentation via Synergistic Angiogenesis, Self-Swelling and Osteogenesis. Bioact. Mater..

[130] Yang X.-Y., Li Y., Lemaire A., Yu J.-G., Su B.-L. (2009). Hierarchically Structured Functional Materials: Synthesis Strategies for Multimodal Porous Networks. Pure Appl. Chem..

[131] Mayr J., Saldías C., Díaz D.D. (2018). Release of Small Bioactive Molecules from Physical Gels. Chem. Soc. Rev..

[132] Barreto M.E., Medeiros R.P., Shearer A., Fook M.V., Montazerian M., Mauro J.C. (2022). Gelatin and Bioactive Glass Composites for Tissue Engineering: A Review. J. Funct. Biomater..

[133] Chen Y.-H., Rao Z.-F., Liu Y.-J., Liu X.-S., Liu Y.-F., Xu L.-J., Wang Z.-Q., Guo J.-Y., Zhang L., Dong Y.-S. (2021). Multifunctional Injectable Hydrogel Loaded with Cerium-Containing Bioactive Glass Nanoparticles for Diabetic Wound Healing. Biomolecules.

[134] Zhou L., Fan L., Zhang F.-M., Jiang Y., Cai M., Dai C., Luo Y.-A., Tu L.-J., Zhou Z.-N., Li X.-J. (2021). Hybrid Gelatin/Oxidized Chondroitin Sulfate Hydrogels Incorporating Bioactive Glass Nanoparticles with Enhanced Mechanical Properties, Mineralization, and Osteogenic Differentiation. Bioact. Mater..

[135] Chen M., Wang Y., Yuan P., Wang L., Li X., Lei B. (2024). Multifunctional Bioactive Glass Nanoparticles: Surface–Interface Decoration and Biomedical Applications. Regen. Biomater..

[136] Fernandes J.S., Gentile P., Pires R.A., Reis R.L., Hatton P.V. (2017). Multifunctional Bioactive Glass and Glass-Ceramic Biomaterials with Antibacterial Properties for Repair and Regeneration of Bone Tissue. Acta Biomater..

[137] Wei S., Ma J.-X., Xu L., Gu X.-S., Ma X.-L. (2020). Biodegradable Materials for Bone Defect Repair. Military Med. Res..

[138] Xu B., Zheng P., Gao F., Wang W., Zhang H., Zhang X., Feng X., Liu W. (2017). A Mineralized High Strength and Tough Hydrogel for Skull Bone Regeneration. Adv. Funct. Mater..

[139] Chang S., Wang J., Xu N., Wang S., Cai H., Liu Z., Wang X. (2022). Facile Construction of Hybrid Hydrogels with High Strength and Biocompatibility for Cranial Bone Regeneration. Gels.

[140] Lu G., Li X., Wang P., Li X., Wang Y., Zhu J., Ronca A., D’Amora U., Liu W., Hui X. (2023). Polysaccharide-Based Composite Hydrogel with Hierarchical Microstructure for Enhanced Vascularization and Skull Regeneration. Biomacromolecules.

[141] Pal P., Tucci M.A., Fan L.W., Bollavarapu R., Lee J.W., Salazar Marocho S.M., Janorkar A.V. (2023). Functionalized Collagen/Elastin-Like Polypeptide Hydrogels for Craniofacial Bone Regeneration. Adv. Healthc. Mater..

[142] Li X., Pang Y., Guan L., Li L., Zhu Y., Whittaker A.K., Yang B., Zhu S., Lin Q. (2024). Mussel-Inspired Antimicrobial Hydrogel with Cellulose Nanocrystals/Tannic Acid Modified Silver Nanoparticles for Enhanced Calvarial Bone Regeneration. Int. J. Biol. Macromol..

[143] Jing X., Xu C., Su W., Ding Q., Ye B., Su Y., Yu K., Zeng L., Yang X., Qu Y. (2023). Photosensitive and Conductive Hydrogel Induced Innerved Bone Regeneration for Infected Bone Defect Repair. Adv. Healthc. Mater..

[144] Yang X., Huang J., Chen C., Zhou L., Ren H., Sun D. (2023). Biomimetic Design of Double-Sided Functionalized Silver Nanoparticle/Bacterial Cellulose/Hydroxyapatite Hydrogel Mesh for Temporary Cranioplasty. ACS Appl. Mater. Interfaces.

[145] You Z., Yu Y., Wang T.H., Wang C., Xiong D., Shi Y., Wang Z., Ye L. (2023). Divalent Anion-Induced Biohydrogels with High Strength, Anti-Swelling, and Bioactive Capability for Enhanced Skull Bone Regeneration. ACS Appl. Mater. Interfaces.

[146] Zhan Y., Yang K., Zhao J., Wang K., Li Z., Liu J., Liu H., Liu Y., Li W., Su X. (2024). Injectable and in situ Formed Dual-Network Hydrogel Reinforced by Mesoporous Silica Nanoparticles and Loaded with BMP-4 for the Closure and Repair of Skull Defects. ACS Biomater. Sci. Eng..

[147] Hu H., Zhang H., Bu Z., Liu Z., Lv F., Pan M., Huang X., Cheng L. (2022). Small Extracellular Vesicles Released from Bioglass/Hydrogel Scaffold Promote Vascularized Bone Regeneration by Transferring miR-23a-3p. Int. J. Nanomed..

[148] Chen M., Zhang Y., Zhang W., Li J. (2020). Polyhedral Oligomeric Silsesquioxane-Incorporated Gelatin Hydrogel Promotes Angiogenesis during Vascularized Bone Regeneration. ACS Appl. Mater. Interfaces.

[149] Du Y., Liu Y., Zhang Y., Nie Y., Xu Z., Qin L., Zhang W., Lai Y. (2025). Structurally and Functionally Adaptive Biomimetic Periosteum: Materials, Fabrication, and Construction Strategies. Wiley Online Libr..

[150] Mellon S.J., Tanner K. (2012). Bone and its Adaptation to Mechanical Loading: A Review. Int. Mater. Rev..

[151] Grujicic M., Arakere G., Xie X., LaBerge M., Grujicic A., Wagner D., Vallejo A. (2010). Design-Optimization and Material Selection for a Femoral-Fracture Fixation-Plate Implant. Mater. Des..

[152] Chen R., Chen H.-B., Xue P.-P., Yang W.-G., Luo L.-Z., Tong M.-Q., Zhong B., Xu H.-L., Zhao Y.-Z., Yuan J.-D. (2021). HA/MgO Nanocrystal-Based Hybrid Hydrogel with High Mechanical Strength and Osteoinductive Potential for Bone Reconstruction in Diabetic Rats. J. Mater. Chem. B.

[153] Céspedes-Valenzuela D.N., Sánchez-Rentería S., Cifuentes J., Gantiva-Diaz M., Serna J.A., Reyes L.H., Ostos C., Cifuentes-De la Portilla C., Muñoz-Camargo C., Cruz J.C. (2021). Preparation and Characterization of an Injectable and Photo-Responsive Chitosan Methacrylate/Graphene Oxide Hydrogel: Potential Applications in Bone Tissue Adhesion and Repair. Polymers.

[154] Ullah I., Hussain Z., Ullah S., ul ain Zahra Q., Zhang Y., Mehmood S., Liu X., Kamya E., Ghani M.W., Mansoorianfar M. (2023). An Osteogenic, Antibacterial, and Anti-Inflammatory Nanocomposite Hydrogel Platform to Accelerate Bone Reconstruction. J. Mater. Chem. B.

[155] Wang Y., Zhu W., Xiao K., Li Z., Ma Q., Li W., Shen S., Weng X. (2019). Self-Healing and Injectable Hybrid Hydrogel for Bone Regeneration of Femoral Head Necrosis and Defect. Biochem. Biophys. Res. Commun..

[156] Peyravian N., Milan P.B., Kebria M.M., Mashayekhan S., Ghasemian M., Amiri S., Hamidi M., Shavandi A., Moghtadaei M. (2024). Designing and Synthesis of Injectable Hydrogel Based on Carboxymethyl Cellulose/Carboxymethyl Chitosan Containing QK Peptide for Femoral Head Osteonecrosis Healing. Int. J. Biol. Macromol..

[157] Liu X., George M.N., Li L., Gamble D., Miller II A.L., Gaihre B., Waletzki B.E., Lu L. (2020). Injectable Electrical Conductive and Phosphate Releasing Gel with Two-Dimensional Black Phosphorus and Carbon Nanotubes for Bone Tissue Engineering. ACS Biomater. Sci. Eng..

[158] Zhang G., Wang X., Meng G., Xu T., Shu J., Zhao J., He J., Wu F. (2023). Enzyme-Mineralized PVASA Hydrogels with Combined Toughness and Strength for Bone Tissue Engineering. ACS Appl. Mater. Interfaces.

[159] Qin B., Dong H., Tang X., Liu Y., Feng G., Wu S., Zhang H. (2023). Antisense yycF and BMP-2 Co-Delivery Gelatin Methacryloyl and Carboxymethyl Chitosan Hydrogel Composite for Infective Bone Defects Regeneration. Int. J. Biol. Macromol..

[160] Hosseini Hooshiar M., Badkoobeh A., Kolahdouz S., Tadayonfard A., Mozaffari A., Nasiri K., Salari S., Safaralizadeh R., Yasamineh S. (2024). The Potential Use of Nanozymes as an Antibacterial Agents in Oral Infection, Periodontitis, and Peri-Implantitis. J. Nanobiotechnol..

[161] Zhang W., Yu M., Cao Y., Zhuang Z., Zhang K., Chen D., Liu W., Yin J. (2023). An Anti-Bacterial Porous Shape Memory Self-Adaptive Stiffened Polymer for Alveolar Bone Regeneration after Tooth Extraction. Bioact. Mater..

[162] Feng P., Zhao R., Tang W., Yang F., Tian H., Peng S., Pan H., Shuai C. (2023). Structural and Functional Adaptive Artificial Bone: Materials, Fabrications, and Properties. Adv. Funct. Mater..

[163] Huang L., Guo Z., Yang X., Zhang Y., Liang Y., Chen X., Qiu X., Chen X. (2025). Advancements in GelMA Bioactive Hydrogels: Strategies for Infection Control and Bone Tissue Regeneration. Theranostics.

[164] Xu S., Hu B., Dong T., Chen B.Y., Xiong X.J., Du L.J., Li Y.L., Chen Y.L., Tian G.C., Bai X.B. (2023). Alleviate Periodontitis and Its Comorbidity Hypertension Using a Nanoparticle-Embedded Functional Hydrogel System. Adv. Healthc. Mater..

[165] Liu J., Liu H., Jia Y., Tan Z., Hou R., Lu J., Luo D., Fu X., Wang L., Wang X. (2022). Glucose-Sensitive Delivery of Tannic Acid by a Photo-Crosslinked Chitosan Hydrogel Film for Antibacterial and Anti-Inflammatory Therapy. J. Biomater. Sci. Polym. Ed..

[166] Yang J., Li R., Wang X., Lu D., Li W., Wang Y. (2025). Mechanically Tunable Fiber-Based Hydrogel Activates PIEZO1–Integrin Axis for Enhanced Bone Repair. J. Nanobiotechnol..

[167] Chang Y.-T., Lai C.-C., Lin D.-J. (2023). Collagen Scaffolds Laden with Human Periodontal Ligament Fibroblasts Promote Periodontal Regeneration in SD Rat Model. Polymers.

[168] Yu Y., You Z., Li X., Lou F., Xiong D., Ye L., Wang Z. (2024). Injectable Nanocomposite Hydrogels with Strong Antibacterial, Osteoinductive, and ROS-Scavenging Capabilities for Periodontitis Treatment. ACS Appl. Mater. Interfaces.

[169] Xu L., Bai X., Yang J., Li J., Xing J., Yuan H., Xie J., Li J. (2020). Preparation and Characterisation of a Gellan Gum-Based Hydrogel Enabling Osteogenesis and Inhibiting Enterococcus faecalis. Int. J. Biol. Macromol..

[170] Medlicott N.J., Rathbone M.J., Tucker I.G., Holborow D.W. (1994). Delivery Systems for the Administration of Drugs to the Periodontal Pocket. Adv. Drug Deliv. Rev..

[171] Li A., Khan I.N., Khan I.U., Yousaf A.M., Shahzad Y. (2021). Gellan Gum-Based Bilayer Mucoadhesive Films Loaded with Moxifloxacin Hydrochloride and Clove Oil for Possible Treatment of Periodontitis. Drug Des. Dev. Ther..

[172] Xu X., Gu Z., Chen X., Shi C., Liu C., Liu M., Wang L., Sun M., Zhang K., Liu Q. (2019). An Injectable and Thermosensitive Hydrogel: Promoting Periodontal Regeneration by Controlled-Release of Aspirin and Erythropoietin. Acta Biomater..

[173] Feng Q., Zhang M., Zhang G., Mei H., Su C., Liu L., Wang X., Wan Z., Xu Z., Hu L. (2024). A Whole-Course-Repair System Based on ROS/Glucose Stimuli-Responsive EGCG Release and Tunable Mechanical Property for Efficient Treatment of Chronic Periodontitis in Diabetic Rats. J. Mater. Chem. B.

[174] Goldring S.R., Goldring M.B. (2016). Changes in the Osteochondral Unit during Osteoarthritis: Structure, Function and Cartilage–Bone Crosstalk. Nat. Rev. Rheumatol..

[175] Guo X., Xi L., Yu M., Fan Z., Wang W., Ju A., Liang Z., Zhou G., Ren W. (2023). Regeneration of Articular Cartilage Defects: Therapeutic Strategies and Perspectives. J. Tissue Eng..

[176] Li Q., Yu H., Zhao F., Cao C., Wu T., Fan Y., Ao Y., Hu X. (2023). 3D Printing of Microenvironment-Specific Bioinspired and Exosome-Reinforced Hydrogel Scaffolds for Efficient Cartilage and Subchondral Bone Regeneration. Adv. Sci..

[177] Jiang G., Li S., Yu K., He B., Hong J., Xu T., Meng J., Ye C., Chen Y., Shi Z. (2021). A 3D-Printed PRP-GelMA Hydrogel Promotes Osteochondral Regeneration through M2 Macrophage Polarization in a Rabbit Model. Acta Biomater..

[178] Lv J., Wu Y., Cao Z., Liu X., Sun Y., Zhang P., Zhang X., Tang K., Cheng M., Yao Q. (2023). Enhanced Cartilage and Subchondral Bone Repair Using Carbon Nanotube-Doped Peptide Hydrogel–Polycaprolactone Composite Scaffolds. Pharmaceutics.

[179] Fang Z., Liu G., Wang B., Meng H., Bahatibieke A., Li J., Ma M., Peng J., Zheng Y. (2024). An Injectable Self-Healing Alginate Hydrogel with Desirable Mechanical and Degradation Properties for Enhancing Osteochondral Regeneration. Carbohydr. Polym..

[180] Cao Y., Zhang H., Qiu M., Zheng Y., Shi X., Yang J. (2024). Biomimetic Injectable and Bilayered Hydrogel Scaffold Based on Collagen and Chondroitin Sulfate for the Repair of Osteochondral Defects. Int. J. Biol. Macromol..

[181] Ji X., Shao H., Li X., Ullah M.W., Luo G., Xu Z., Ma L., He X., Lei Z., Li Q. (2022). Injectable Immunomodulation-Based Porous Chitosan Microspheres/HPCH Hydrogel Composites as a Controlled Drug Delivery System for Osteochondral Regeneration. Biomaterials.

[182] Wu T., Yu S., Chen D., Wang Y. (2017). Bionic Design, Materials and Performance of Bone Tissue Scaffolds. Materials.

[183] Guan Q.-F., Han Z.-M., Zhu Y., Xu W.-L., Yang H.-B., Ling Z.-C., Yan B.-B., Yang K.-P., Yin C.-H., Wu H. (2021). Bio-Inspired Lotus-Fiber-Like Spiral Hydrogel Bacterial Cellulose Fibers. Nano Lett..

[184] Han X., Sun M., Chen B., Saiding Q., Zhang J., Song H., Deng L., Wang P., Gong W., Cui W. (2021). Lotus Seedpod-Inspired Internal Vascularized 3D Printed Scaffold for Bone Tissue Repair. Bioact. Mater..

[185] Wang X., Fang J., Zhu W., Zhong C., Ye D., Zhu M., Lu X., Zhao Y., Ren F. (2021). Bioinspired Highly Anisotropic, Ultrastrong and Stiff, and Osteoconductive Mineralized Wood Hydrogel Composites for Bone Repair. Adv. Funct. Mater..

[186] Gao B., Ni H., Lai J., Gao N., Luo X., Wang Y., Chen Y., Zhao J., Yu Z., Zhang J. (2025). Macrophage Response to Fibrin Structure Mediated by Tgm2-Dependent Mitochondrial Mechanosensing. Bioact. Mater..

[187] Chen J., Chen J., Zhu Z., Sun T., Liu M., Lu L., Zhou C., Luo B. (2022). Drug-Loaded and Anisotropic Wood-Derived Hydrogel Periosteum with Super Antibacterial, Anti-Inflammatory, and Osteogenic Activities. ACS Appl. Mater. Interfaces.

[188] Dou Y., Wang Z.-P., He W., Jia T., Liu Z., Sun P., Wen K., Gao E., Zhou X., Hu X. (2019). Artificial Spider Silk from Ion-Doped and Twisted Core-Sheath Hydrogel Fibres. Nat. Commun..

[189] Wu S., Liu Z., Gong C., Li W., Xu S., Wen R., Feng W., Qiu Z., Yan Y. (2024). Spider-Silk-Inspired Strong and Tough Hydrogel Fibers with Anti-Freezing and Water Retention Properties. Nat. Commun..

[190] Yang K., Zhou D., Wang Y., Chen R., Dong Q., Xiao P., Zhou Y., Zhang J. (2025). Spider Silk-Inspired Hyaluronic Acid-Based Hydrogels with Superior Self-Healing Capability and Enhanced Strength. ChemSusChem.

[191] Zhang Z., Ma Y., Yu B., Ma S., Yang H., Liu L., Yu J., Pei X., Cai M., Zhou F. (2024). Vertical and Horizontal Double Gradient Design for Super-Slippery and High-Bearing Hydrogel Skeleton. Adv. Funct. Mater..

[192] Chen Q., Zhang X., Chen K., Feng C., Wang D., Qi J., Li X., Zhao X., Chai Z., Zhang D. (2022). Bilayer Hydrogels with Low Friction and High Load-Bearing Capacity by Mimicking the Oriented Hierarchical Structure of Cartilage. ACS Appl. Mater. Interfaces.

[193] Waresindo W.X., Luthfianti H.R., Priyanto A., Hapidin D.A., Edikresnha D., Aimon A.H., Suciati T., Khairurrijal K. (2023). Freeze–Thaw Hydrogel Fabrication Method: Basic Principles, Synthesis Parameters, Properties, and Biomedical Applications. Mater. Res. Express.

